# Scientometric analysis of glioblastoma and blood-brain barrier research (1995−2024): evolving trends and therapeutic challenges

**DOI:** 10.3389/fonc.2025.1649414

**Published:** 2025-09-25

**Authors:** Xuan-Hao Pan, Nan Wang, Yi-Chun He, Jing Su, Yu-Fei Gao

**Affiliations:** ^1^ Department of Neurosurgery, China−Japan Union Hospital of Jilin University, Changchun, Jilin, China; ^2^ Jilin Province Neuro-oncology Engineering Laboratory, Changchun, Jilin, China; ^3^ Jilin Provincial Key Laboratory of Neuro-oncology, Changchun, Jilin, China; ^4^ Department of Pathophysiology, College of Basic Medical Sciences, Jilin University, Changchun, Jilin, China

**Keywords:** glioblastoma, blood-brain barrier, research trends, scientometric analysis, drug delivery

## Abstract

**Background:**

Glioblastoma (GBM) is one of the most aggressive neurogenic tumors. Despite advances in treatment, the presence of the blood-brain barrier (BBB) continues to pose significant challenges to effective therapeutic delivery. However, to date, no comprehensive bibliometric analysis has systematically evaluated the relationship between GBM and the BBB over the past three decades.

**Objective:**

This study provides an overview of research progress on GBM and the BBB, with emphasis on structural and functional changes of the BBB. It also identifies current research hotspots, predicts emerging trends, and offers insights for future investigations. Method: Literature from the past 30 years was retrieved from the Web of Science Core Collection and PubMed. Bibliometric analysis was performed using the R programming language, and data visualization was conducted with VOSviewer, CiteSpace, and Tableau.

**Result:**

Since 2015, publication output and academic influence in this field have increased exponentially. The United States leads in both publication volume and citation count, and engages in extensive international collaborations. *Cancer Research*, the leading journal in this field, ranks first with 6,775 citations over the past 30 years. Keyword analysis reveals that the field has primarily focused on tumor-associated angiogenesis, the role of vascular endothelial growth factor (VEGF) in BBB disruption, optimization of drug delivery strategies, the influence of the tumor microenvironment (TME) on tumor progression, and advances in precision medicine. Co-citation analysis, citation burst detection, and Latent Dirichlet Allocation (LDA) topic modeling have identified seminal publications and key developmental trajectories. Notably, a comprehensive analysis of clinical trial literature revealed a gradual shift in research focus from traditional morphological and single-agent efficacy studies to more integrated approaches, including BBB permeability regulation, targeted drug delivery, and multimodal functional imaging.

**Conclusion:**

This study offers a comprehensive overview of GBM−BBB research trends over the past 30 years. It advances the understanding of their interplay and provides theoretical guidance for overcoming the BBB and improving GBM outcomes.

## 1 Introduction

GBM is one of the most common and aggressive malignant tumors of the central nervous system, with a two-year overall survival rate of approximately 25% and a five-year survival rate of only 7.2% (1, 2). Although molecular pathology has led to new cancer therapies, GBM patients have gained limited benefit, as many experimental drugs have failed in clinical trials. The dismal prognosis of GBM is, to a large extent, closely related to the structural and functional characteristics of the BBB.

The core structural components of the BBB are the vascular endothelial cells (ECs), which are non-fenestrated and connected by tight junctions (TJs) that seal the paracellular space between adjacent endothelial cells, forming a continuous and selective barrier along the vascular lumen. This architecture results in a high transendothelial electrical resistance (1500−2000 Ω·cm²), which supports the maintenance of EC polarity and cellular adhesion, while effectively restricting the paracellular diffusion of macromolecules (3). In addition, the BBB expresses a variety of transport proteins that selectively permit the translocation of specific substances (4). It also strictly limits the passage of immune cells, particularly lymphocytes. These characteristics endow the BBB with multiple functions, including serving as a physical barrier, a transport barrier, and an immunological barrier (5). Under the restrictive influence of the BBB, temozolomide (TMZ) chemotherapy, one of the standard treatments for GBM, achieves a drug concentration in tumor tissue that is only approximately 20% of the systemic level, far below the ideal therapeutic threshold. According to findings from the NABTT CNS Consortium, among 365 patients who received combined radiotherapy and temozolomide treatment (RT + TMZ), the median survival was 14.4 months, with a one-year survival rate of 40% and a two-year survival rate of 20−25% (6).

Although most GBM patients exhibit varying degrees of BBB disruption within tumor regions, which can be visualized on MRI using gadolinium-based contrast agents (7), intact portions of the BBB often remain within the tumor. MRI findings further suggest that the T2/FLAIR regions beyond contrast-enhanced areas, which are highlighted by ^18^F-DOPA tracer uptake, may still harbor tumor burden (8). Due to the structural integrity of the BBB in these regions, transporter proteins such as P-glycoprotein (P-gp), multidrug resistance-associated protein 4 (MRP4), and breast cancer resistance protein (BCRP) remain functionally active on the luminal membrane of brain capillary endothelial cells, where they mediate active efflux (9–11). This results in reduced intratumoral drug permeability and subtherapeutic concentrations, contributing to GBM recurrence. Overcoming the restrictive properties of these intact barriers and enhancing drug penetration across the BBB are therefore critical for improving therapeutic outcomes in GBM.

In recent years, research interest in the structural and functional aspects of the BBB in the context of GBM has grown significantly. There is an urgent need for a comprehensive review and analysis of the existing literature to better understand the past achievements and breakthroughs in this area, as well as their implications for the treatment of GBM. To address this need, we applied mathematical and statistical techniques to evaluate and quantify the literature in this field. To this end, we conducted a bibliometric analysis of relevant publications from the past 30 years to map the evolution of this field, identify research hotspots, and highlight emerging trends.

## 2 Materials and methods

### 2.1 Data source and literature search

The raw data analyzed in this study were obtained from two databases: the Web of Science (WoS) Core Collection, which includes the Science Citation Index Expanded (SCI-E) and the Social Sciences Citation Index (SSCI), and PubMed. WoS is widely recognized as a reliable platform for bibliometric analysis. To ensure comprehensive and accurate retrieval, a truncation-based search strategy using wildcards (e.g., *) was applied in WoS. The search query used was: ((TS=((“glioma*” OR “glioblastoma*”))) AND TS=((“blood-brain barrier”))) AND TS=((structure* OR function* OR remodel* OR disrupt* OR impair* OR alter*)).

However, since PubMed does not support wildcard truncation, an equivalent search strategy using fully spelled-out terms was implemented to maintain semantic consistency across databases. This two-database approach ensured comprehensive coverage of both basic research and clinical trial literature for further analysis (see **Supplementary Material**, **Supplementary Table S1**).

Given the inherent differences between WoS and PubMed in terms of indexing standards, clinical tagging systems, and metadata structures, directly merging data from both sources could compromise the comparability and scientific validity of the bibliometric results. Therefore, this study adopted a “separate database analysis plus integrated discussion” approach to independently explore the research structures and thematic trends of basic and clinical studies, respectively.

### 2.2 Data screening

WoS data were mainly used to track the evolution of basic and high-impact research, while PubMed data focused on analyzing clinical trial literature and intervention strategies, serving as a supplement to evaluate translational potential and clinical implementation strategies.

#### 2.2.1 Inclusion criteria

In terms of document types, the WoS search was restricted to English-language “Articles” and “Reviews”, while the PubMed search was refined to include studies categorized as “Clinical Trials”. The search covered the period from 1995 to 2024 and included only English-language publications.

#### 2.2.2 Exclusion criteria

The following records were excluded from the analysis: (1) publications including, but not limited to, proceeding papers, meeting abstracts, editorial materials, book chapters, letters, retracted publications, correction notices, and publications with expressions of concern; (2) duplicate records; and (3) publications with incomplete or missing bibliographic information. The inclusion and exclusion processes were independently conducted by two reviewers. In cases of disagreement, a third reviewer was consulted to resolve discrepancies and reach a consensus.

#### 2.2.3 Data standardization

All searches were conducted on the same day to ensure temporal consistency. The retrieved records were exported in plain text format, and preprocessing involved the removal of special characters and redundant whitespace. To enhance consistency and reproducibility in the bibliometric analysis, a structured keyword standardization protocol was implemented. First, all extracted keywords were cleaned to eliminate typographical errors, unnecessary punctuation, and spacing anomalies. Subsequently, synonymous terms were standardized and merged using a combined approach involving ontology referencing (e.g., MeSH vocabulary), keyword co-occurrence patterns, and manual curation (see **Supplementary Material**, **Supplementary Table S2**). For example, “glioblastoma multiforme” and “GBM” were unified under the standard term “glioblastoma”, while “barrier disruption” and “barrier permeability” were standardized as “Blood-Brain Barrier Disruption” and “Blood-Brain Barrier Permeability”, respectively, to ensure consistency in structural and semantic representation. To ensure consistency in geographical analysis, country and region names were also standardized. For instance, “Hong Kong”, “Macau”, and “Taiwan” were categorized under “China”.

### 2.3 Data analysis and visualization

This study utilized several software tools for data analysis and visualization, including CiteSpace 6.4 R1 (64-bit), VOSviewer 1.6.20, and the Bibliometrix online analysis platform (http://bibliometric.com/). These tools were used to analyze co-cited references, keyword co-occurrence, country-level contributions, institutional affiliations, author collaborations, journal impact, and citation bursts.

The Bibliometrix online analysis platform was used to examine annual publication outputs in GBM and BBB research and to identify the most productive countries and influential authors. This analysis revealed the major contributors in terms of scientific output and highlighted their positions within the global research network. In addition, we analyzed citation patterns of high-impact journals to identify core journals with significant academic influence in this field. All analytical results were visualized using Tableau, allowing for intuitive representation of annual publication trends, country-level output, author productivity, and journal impact, thereby providing deeper insights into research hotspots and emerging trends.

VOSviewer was employed to construct a scientific knowledge network and to visualize the evolution of collaboration among authors, institutions, and countries. In VOSviewer, each node represents an entity, while connecting lines indicate collaborative relationships. The thickness of the lines reflects the strength of the collaboration, while the size of the nodes corresponds to research output and academic impact. This analysis provides a clear visualization of the core entities with strong collaboration and highlights their key roles within the global research network.

CiteSpace was utilized to perform citation burst detection for both keywords and references, revealing research topics and publications that experienced significant growth during specific time periods. This analysis enabled us to identify research hotspots and predict potential future directions concerning the relationship between GBM and the BBB in terms of structure and function. Additionally, the co-occurrence analysis feature in CiteSpace was used to construct networks of keywords and references. These networks enabled the analysis of their temporal evolution and intuitively illustrated peak activity periods and thematic developments. Timeline views and mountain maps were also generated. The visual analysis of keywords and references further involved several important indicators: Centrality measures the importance of a keyword or reference within the network, identifying bridging nodes across domains that occupy crucial positions in the overall research framework. The modularity value (Q value) evaluates the degree of clustering within the network; a higher Q value indicates a clearer and more meaningful modular structure. Q values range from 0 to 1, and a value greater than 0.3 suggests that the clustering structure is statistically significant. The silhouette value (S value) assesses the homogeneity of clusters within the network. An S value closer to 1 indicates higher internal consistency, and values above 0.7 are generally considered to represent highly reliable clustering results.

**Figure 1:**
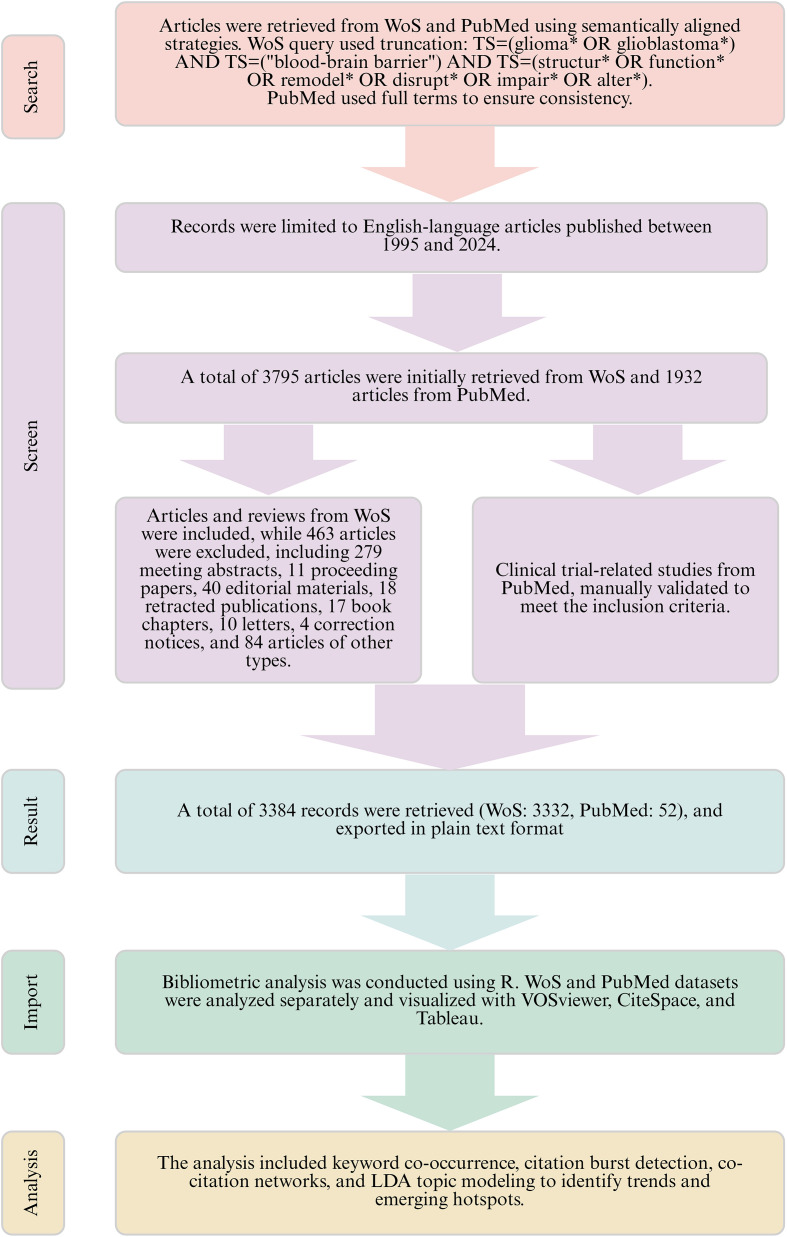
Flowchart for data collection and analysis of the relationship between GBM and BBB structure and function.

In addition, we used R to conduct a comprehensive analysis of clinical trial literature. The text data were first preprocessed by removing stop words, punctuation, and low-frequency terms to enhance the accuracy of text mining. Based on the cleaned corpus, an LDA topic model was constructed using the LDA() function from the topic models package to identify major research themes and examine their temporal evolution. Furthermore, the included studies were categorized by clinical trial type (Interventional Clinical Trial vs. Observational Clinical Study) and research content type (Mechanistic Studies, Efficacy and Safety Assessment, Diagnostic Imaging Studies). A keyword co-occurrence network was also generated to visualize the associations between different intervention strategies, thereby uncovering their structural relationships and supporting a coordinated evaluation of translational research between basic science and clinical practice. The Experimental Flowchart is shown in **Figure 1**.

## 3 Results

### 3.1 Publication trends and global distribution of research outputs

**Figure 2:**
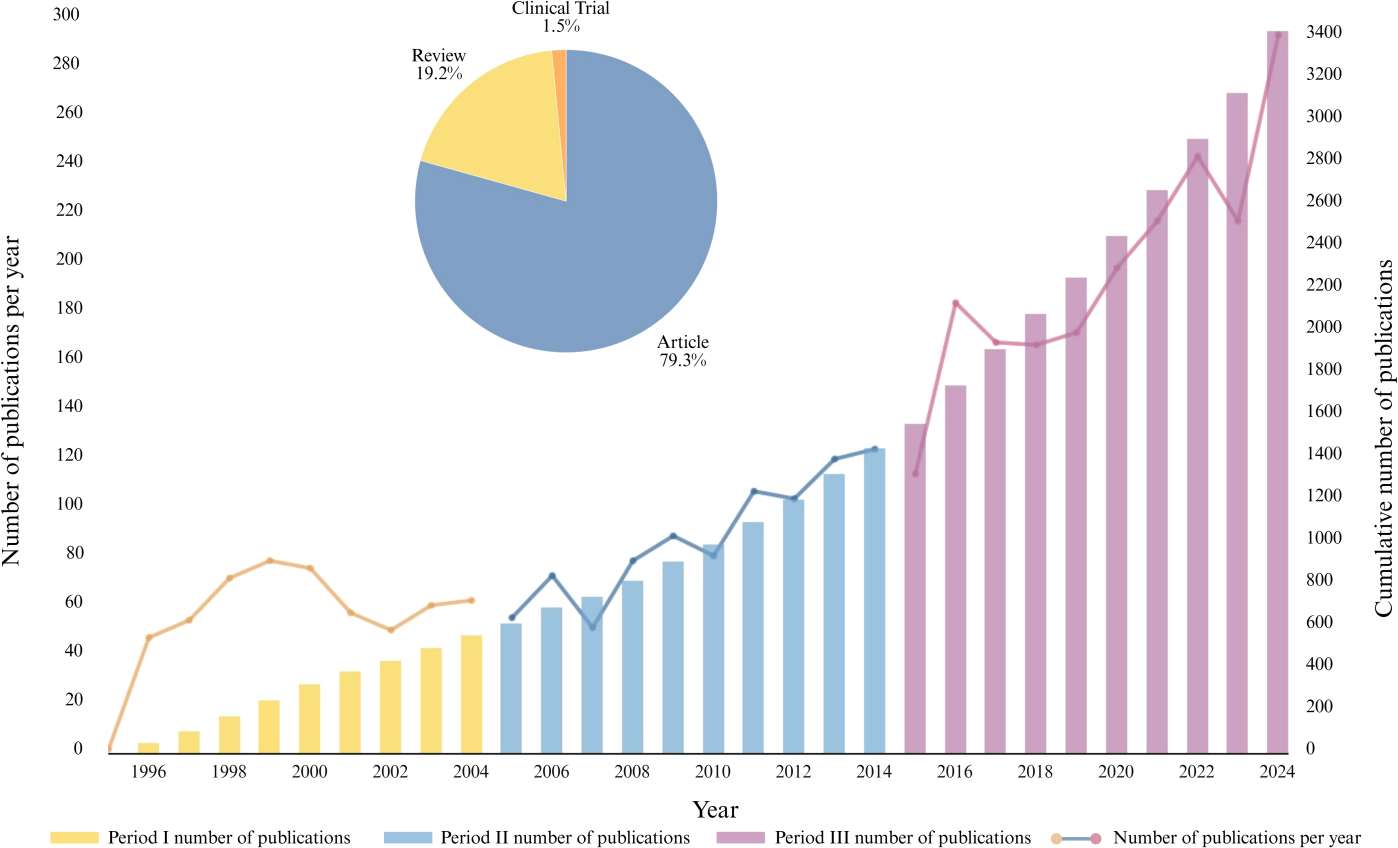
Annual and cumulative publication trends and publication type distribution in GBM and BBB research (1995−2024).

We retrieved publications related to the structural and functional aspects of GBM and the BBB from both the Web of Science Core Collection and PubMed, covering the period from 1995 to 2024. **Figure 2** illustrates the annual publication volume and cumulative total from both the Web of Science and PubMed databases over the past three decades. Overall, the number of research articles, reviews (from WoS), and clinical trial-related publications (from PubMed) in this field has shown a steady upward trend. Specifically, the growth in publication output can be broadly divided into three developmental phases.

Phase I (1995−2004): During this initial stage, research on the interaction between GBM and the BBB was still in its infancy. The primary focus was on the fundamental understanding of GBM and preliminary pathological investigations. A total of 556 publications were produced during this period, with an average of approximately 50 articles published per year.

Phase II (2005−2014): This period marked significant progress in the field, with increasing attention directed toward BBB-related research. The average annual number of publications approached 100, with a total of 875 articles published during this decade.

Phase III (2015−2024): During this period, the annual number of publications experienced exponential growth, reaching a total of 1,953 articles. The trend line on the right side of **Figure 2** clearly illustrates this surge. Since 2015, there has been a substantial increase in both research activity and the number of publications related to GBM and the BBB, indicating that this topic has gradually become a major focus within the academic community. An increasing number of researchers have entered the field, contributing to several breakthrough developments. Except for **Figure 2**, which integrates data from both the Web of Science and PubMed, all subsequent analyses in Sections 3.3 to 3.7 are based exclusively on the Web of Science Core Collection.

**Figure 3:**
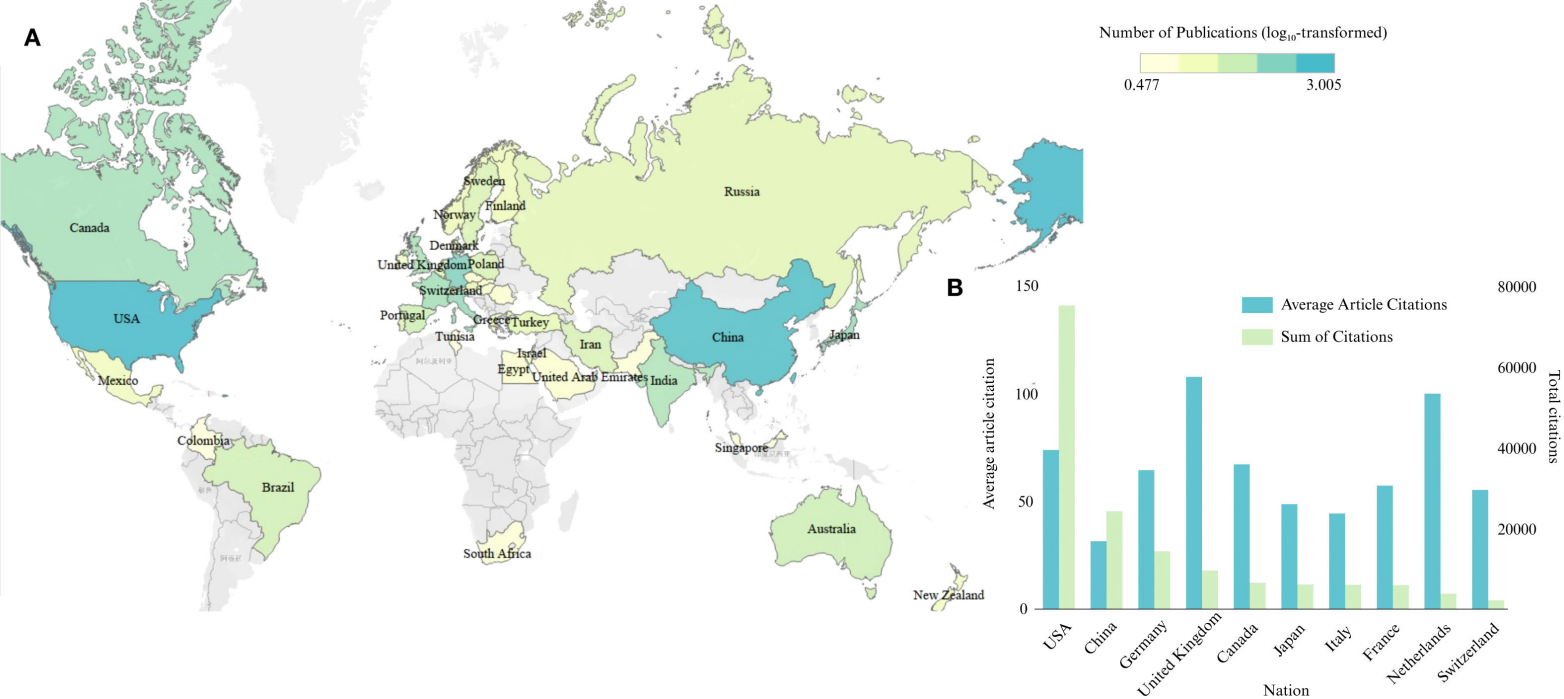
Distribution of publication output and citation impact by country. **(A)** Geographic heatmap of countries involved in the research; color intensity represents the number of publications (log_10_-transformed). **(B)** Total citations and average citations per article for the top 10 countries.

**Figure 3A** shows that the United States (1,012 publications), China (764 publications), Germany (225 publications), the United Kingdom (92 publications), and Canada (102 publications) are among the leading countries in research on the impact of GBM on the BBB. By contrast, most other Asian countries, along with regions in South America and Africa, have made relatively modest contributions to this field. Further insights from **Figure 3B** indicate that the United States remains the most prominent contributor to GBM and the BBB, with the highest number of publications and total citations. Although China has contributed a substantial number of publications and total citations, its average citations per article remain relatively low. The United Kingdom and Germany, despite having fewer publications, exhibit higher average citation counts per article, suggesting strong research quality and widespread academic recognition. In addition, countries such as Japan, France, and the Netherlands have also contributed to this field, although their overall scientific impact remains moderate.

### 3.2 Analysis of core authors and journals

**Figure 4:**
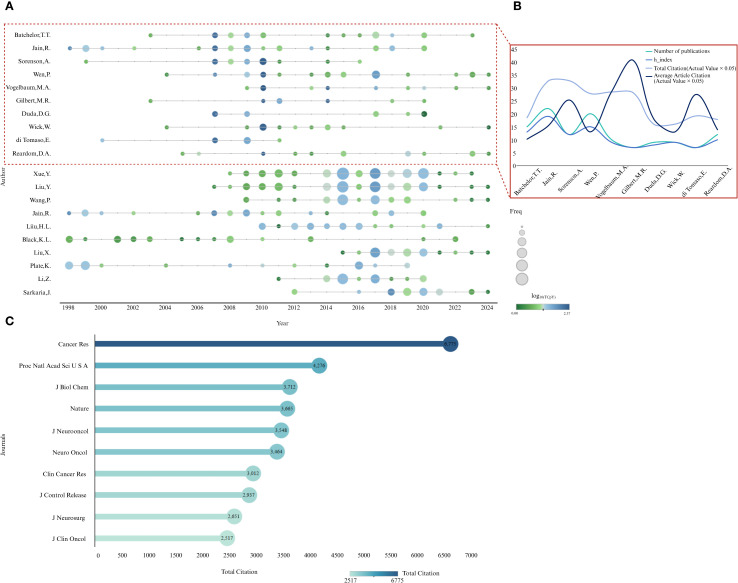
Publication and citation trends of high-impact and high-productivity authors. **(A)** Annual publication counts and citations per year for the top 10 authors. Bubble size represents the number of publications per year, and color intensity indicates the number of citations per year. **(B)** Publication counts, h-index, total citations (scaled by 0.05), and average annual citations (scaled by 0.05) for the top 10 high-impact authors. **(C)** Total citations and publication impact of journals.

**Figure 4** presents the publication and citation metrics of the top 10 high-impact and high-output authors in this field. As shown in **Figure 4A**, the publication and citation trajectories of high-impact authors show noticeable variability, with their academic influence predominantly concentrated between 2005 and 2015. In contrast, most highly productive authors published the majority of their work between 2015 and 2020. **Figure 4B** displays a line chart summarizing the top 10 high-impact authors, including metrics such as number of publications, h-index, total citations (actual value × 0.05), and average article citations (actual value × 0.05). Although authors such as Vogelbaum, M.A. and Gilbert, M.R. do not rank highly in terms of h-index, they exhibit relatively high average citations per article, suggesting a strong impact per publication.

**Figure 4C** presents the top 10 most influential journals in the field of GBM and BBB research. *Cancer Research* ranks first with 6,775 citations, exhibiting a citation impact several times greater than any of the other journals listed. It is followed by *Proceedings of the National Academy of Sciences USA* (4,276 citations), *Journal of Biological Chemistry* (3,712 citations), and *Nature* (3,665 citations). These journals exert substantial influence in the field, and articles published in them typically receive high recognition within the academic community. These results were exported directly from Biblioshiny (Bibliometrix) and visualized in Tableau.

### 3.3 Collaboration network analysis

**Figure 5:**
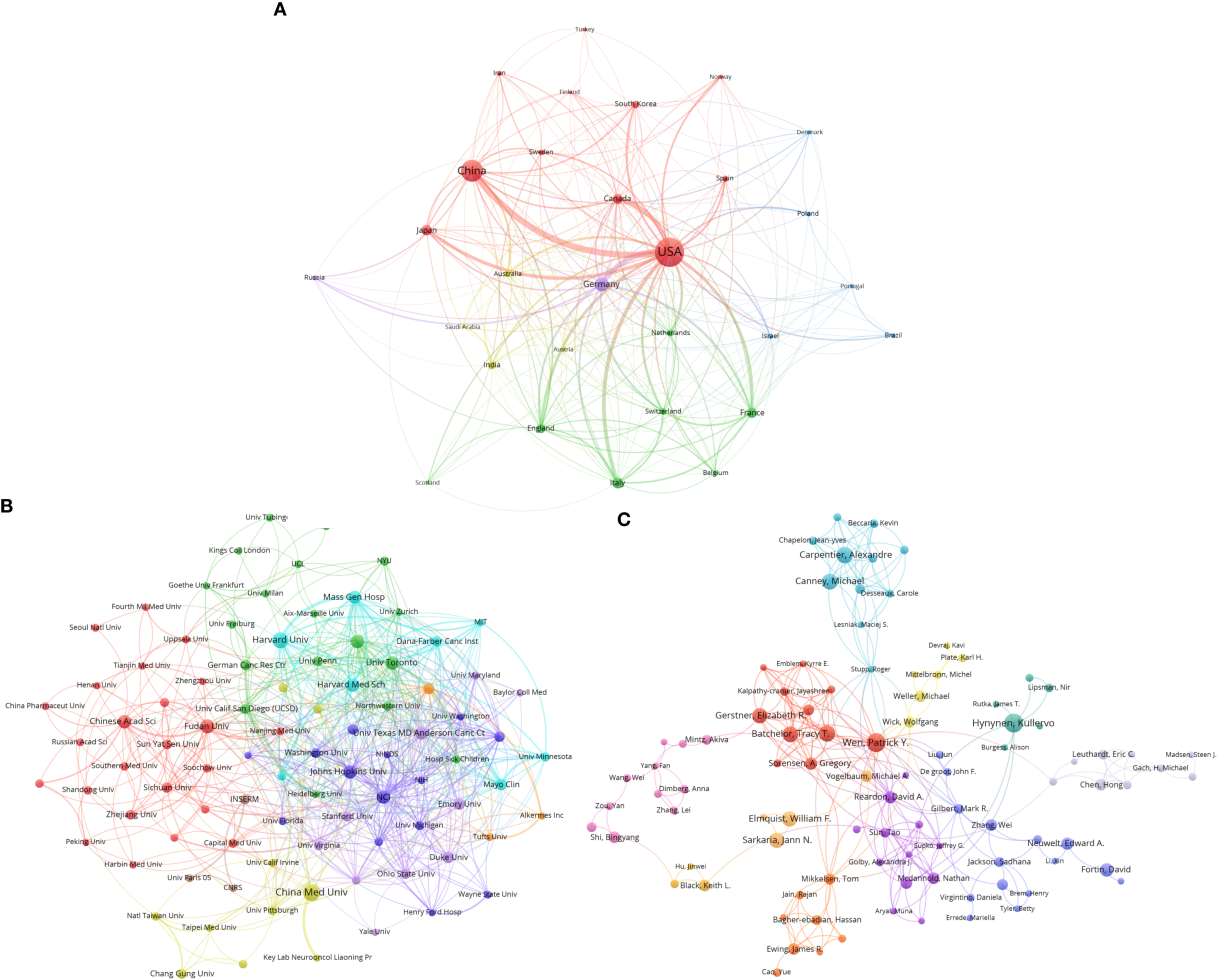
Collaboration networks at three levels in GBM and BBB research. **(A)** International collaboration network. **(B)** Institutional collaboration network. **(C)** Author collaboration network. Nodes represent entities at each level, with size indicating publication volume. Edges represent collaborative relationships, with line thickness reflecting collaboration strength. Colors indicate collaboration clusters.

From 1995 to 2024, authors from 79 countries published articles on GBM and the BBB in international journals. We imported the downloaded records (plain-text format) into VOSviewer, set Type of analysis to “Co-authorship” and Unit of analysis to “Countries,” and applied a publication threshold of ≥16 documents, identifying 30 countries for the collaboration analysis. As shown in **Figure 5A**, China has established close research collaborations with several countries, particularly the United States, selected European nations, and South Korea. In contrast, the United States exhibits a broader and more integrated collaboration network, maintaining strong ties not only with European countries such as France, Germany, and Italy, but also with Asian countries such as China, Japan, and South Korea. This highlights the United States’ leading role in global scientific collaboration and its proactive engagement in international research initiatives. In addition, France, Germany, and Italy have also collaborated extensively with China and the United States, thereby fostering sustained cross-regional cooperation and promoting knowledge exchange in this domain.

Using VOSviewer, we set Type of analysis to “Co-authorship” and Unit of analysis to “Institutions”, and applied a publication threshold of ≥15 documents, identifying 90 institutions for inclusion in the institutional collaboration analysis. A collaboration network was generated using the organization analysis function. As shown in **Figure 5B**, there are notable differences in collaboration patterns among leading research institutions in the field of GBM and BBB, both domestically and internationally.

Specifically, in China, the five most influential institutions (by citation count) are Fudan University (Citations: 4,087; Total Link Strength (TLS): 28), Chinese Academy of Sciences (Citations: 2,934; TLS: 34), China Medical University (Citations: 2,460; TLS: 28), Sichuan University (Citations: 1,416; TLS: 25), and National Taiwan University (Citations: 1,079; TLS: 15). These institutions are densely interconnected, indicating strong domestic collaboration but comparatively sparse international links.

In the red and purple clusters, which represent top-tier global research centers, we observe Harvard University (Citations: 13,564; TLS: 80), Massachusetts General Hospital (Citations: 10,728; TLS: 80), Mayo Clinic (Citations: 8,479; TLS: 50), University of Texas MD Anderson Cancer Center (Citations: 7,798; TLS: 65), and Cleveland Clinic (Citations: 6,641; TLS: 30). Among these, Harvard University, Massachusetts General Hospital, and MD Anderson not only exhibit very high citation impact but also maintain highly integrated collaboration networks. Their high connectivity underscores their national prominence and highlights their global academic influence. In contrast, some Chinese institutions show relatively limited international collaboration and academic impact and tend to occupy more peripheral positions within the global network (see **Supplementary Material**, **Supplementary Table S3** and **Supplementary Figure S1**).

We further constructed an author collaboration network using VOSviewer, set Type of analysis to “Co-authorship” and Unit of analysis to “Authors, “ and set the inclusion threshold at more than six publications. A total of 169 authors met this criterion and were included in the analysis. Using the Author analysis function, a collaboration network was generated. As shown in **Figure 5C**, the field of GBM and BBB research exhibits a complex web of academic collaborations among different authors. The overall structure of the network is characterized by a multi-core, clustered pattern.

Within the network, the purple cluster centered around Patrick Y. Wen (19 documents, 5,403 citations) is partially connected to other collaboration clusters. Patrick Y. Wen ranks among the most highly connected authors in the network and has formed close collaborations with several researchers, including David A. Reardon, Tracy T. Batchelor, and Michael Vogelbaum. This suggests that Patrick Y. Wen is a key contributor in this field, with a highly active and collaborative research team likely involved in leading multiple large-scale projects. Notably, although Jann N. Sarkaria and Kullervo Hynynen demonstrate considerable influence in the field (as indicated by their large node sizes), their collaboration networks appear relatively concentrated. This suggests a stronger focus on intra-group collaboration, with limited involvement in broader cross-team interactions. Similarly, certain peripheral authors or smaller research groups, such as those associated with Akiva Mintz and Eric C. Leuthardt in the pink or gray clusters, exhibit a degree of influence, but their collaborations remain loosely connected and are largely confined to smaller, more independent circles.

### 3.4 Keyword analysis and thematic evolution

Keyword co-occurrence was analyzed in CiteSpace. Time slicing was 1995−2024 (1 year per slice). Node type: Keyword. Link strength: Cosine (default). Scope: Within slices (default). Selection criterion: Top-N per slice = 50. Pruning: Pathfinder, Pruning sliced networks, and Pruning the merged network were enabled. The merged network contained 421 nodes and 2,520 links (network density = 0.0285). The dataset was then exported, and the top 50 keywords by co-occurrence frequency were presented in a table (see **Supplementary Material**, **Supplementary Figure S2**). Apart from the search terms “Glioblastoma” and “Blood-Brain Barrier,” the ten most frequently occurring keywords were: “Gene Expression” (507 occurrences), “*In Vitro*” (486 occurrences), “Angiogenesis” (410 occurrences), “Vascular Permeability” (377 occurrences), “Endothelial Cells” (295 occurrences), “Drug Delivery” (261 occurrences), “Tumor Growth” (249 occurrences), “Cells” (224 occurrences), “Endothelial Growth Factor” (216 occurrences), and “Adjuvant Temozolomide” (185 occurrences). These keywords exhibited strong and well-organized co-occurrence relationships, forming a multi-centered, broad-themed distribution pattern that reinforces the thematic focus of this study.

**Figure 6:**
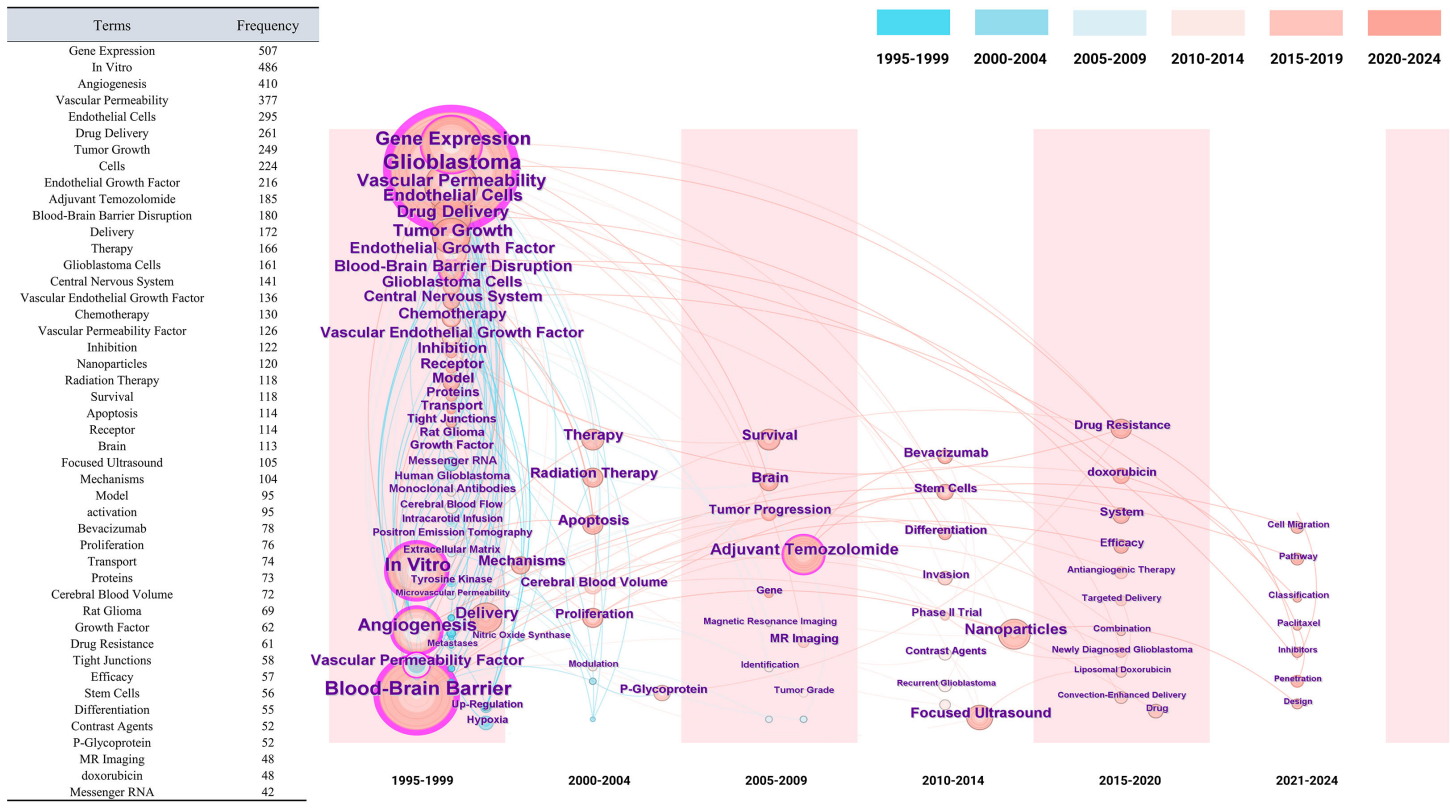
Keyword co-occurrence and temporal distribution analysis in GBM and BBB research. The time-zone diagram illustrates changes in high-frequency keywords over time, with node size representing term frequency, color intensity indicating co-occurrence strength, and connections showing clustering relationships. The accompanying table lists the top 50 keywords by co-occurrence frequency and highlights thematic trends over time.

Using the Timeline View function in CiteSpace, a keyword timeline map was generated to better illustrate the temporal trajectory and evolution of key terms within each cluster. The time slicing was set to five-year intervals, while other parameters remained unchanged. By identifying high-frequency and high-centrality keywords, the analysis reveals major research hotspots and emerging trends. As shown in **Figure 6**, based on the temporal distribution of keywords, research related to GBM and the BBB over the past three decades can be divided into three distinct stages.

From 1995 to 2004, research primarily focused on the fundamental biological mechanisms linking GBM and the BBB, as well as the exploration of targeted therapeutic strategies. Specifically, early studies (1995−1999) concentrated on vascular permeability, BBB disruption, and endothelial growth factor, revealing that GBM induces angiogenesis and upregulates VEGF. These findings demonstrated that VEGF expression is closely linked to tumor vascularization, cerebral edema, and necrosis, and that tumor cells disrupt the BBB via VEGF secretion to facilitate tumor progression. Between 2000 and 2004, the research focus shifted toward more detailed mechanisms and drug delivery strategies. Keywords such as “Mechanisms,” “Apoptosis,” and “Radiation Therapy” reflected a growing emphasis on the functional role of VEGF. Researchers also began investigating the potential of Epidermal Growth Factor Receptor (EGFR) and VEGF inhibitors to penetrate the BBB and improve drug delivery efficiency.

From 2005 to 2014, research primarily focused on optimizing therapeutic strategies and developing novel drug delivery approaches. Between 2005 and 2009, the combination of chemotherapy and radiotherapy emerged as the mainstream treatment strategy for GBM and was shown to significantly improve clinical outcomes. In addition, magnetic resonance imaging (MRI) and cerebral blood flow measurements were widely applied for monitoring tumor progression and evaluating prognosis. Cerebral blood flow, in particular, has been recognized as a potential indicator of tumor invasiveness and patient prognosis, as it is closely associated with tumor growth and may substantially influence the intratumoral distribution and therapeutic efficacy of chemotherapeutic agents. After 2010, the emergence of nanoparticle technology and focused ultrasound (FUS) significantly enhanced drug delivery efficiency. FUS, in particular, enabled the temporary and reversible opening of the BBB, thereby improving drug penetration. Meanwhile, VEGF inhibitors, such as bevacizumab, gradually became a research hotspot and demonstrated promising therapeutic effects in clinical trials.

Between 2015 and 2024, research has increasingly focused on modulation of the TME, mechanisms of drug resistance, precision delivery technologies, and the emergence of novel therapeutic strategies. Drug resistance has become a critical challenge, with particular attention given to anthracycline chemotherapeutics such as doxorubicin and its liposomal formulation (liposomal doxorubicin), which have been investigated for their potential to penetrate the BBB and mitigate drug resistance. Keywords such as “Targeted Delivery,” “Convection-Enhanced Delivery,” “Cell Migration,” “Pathway,” and “Classification” reflect deeper exploration into GBM cell heterogeneity and mechanisms of invasion. This phase also highlights a shift toward leveraging single-cell technologies, tissue sampling, and molecular profiling to uncover the cellular heterogeneity and molecular characteristics of GBM, thereby advancing the development of personalized treatment strategies.

**Figure 7:**
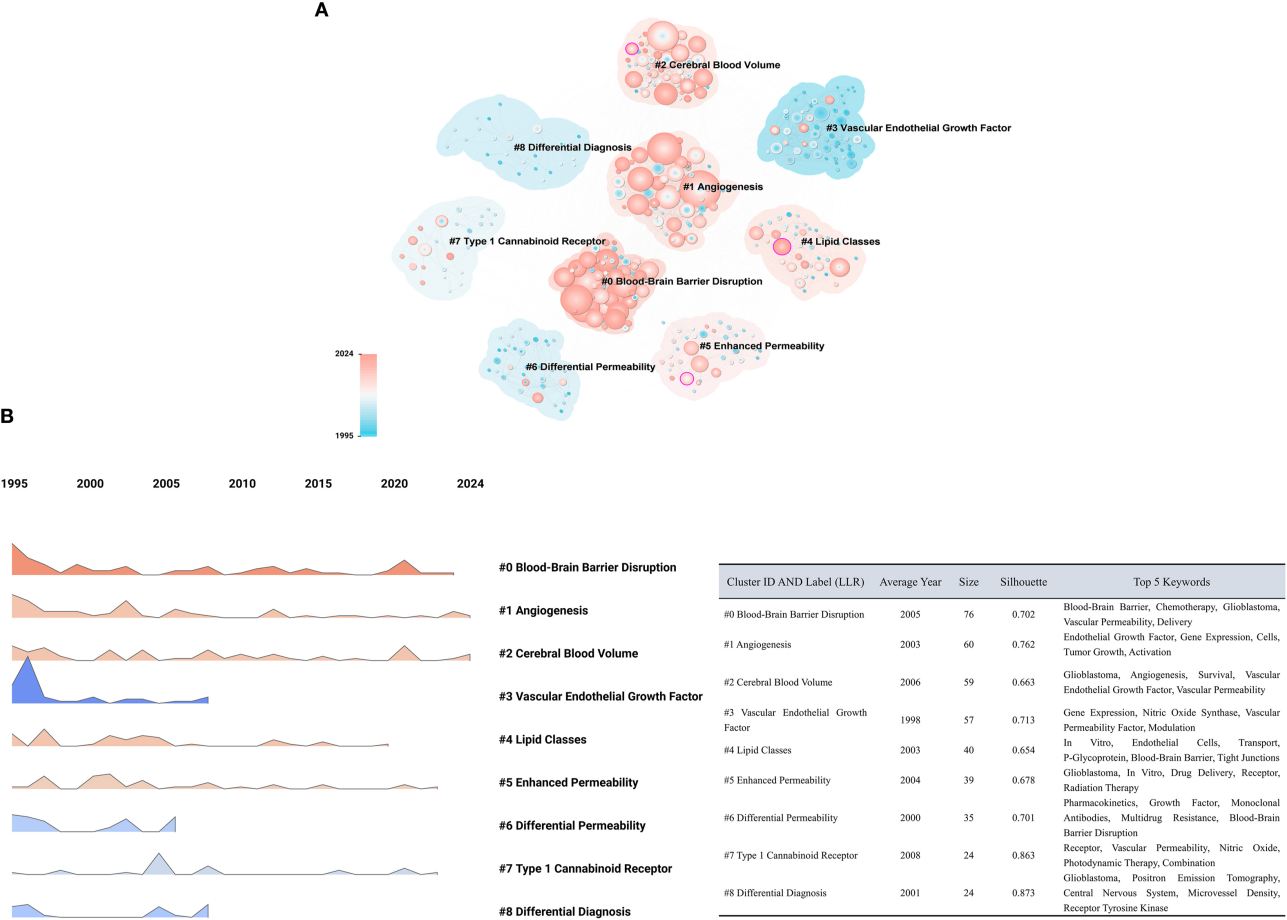
Keyword clustering and temporal trend analysis with cluster characteristics. **(A)** Keyword clustering network. Node size indicates keyword frequency, color intensity reflects significance, and cluster numbering is inversely proportional to cluster size. **(B)** Temporal trends of keyword clusters. Temporal distribution of major keyword clusters; ridge height indicates the publication frequency of keywords within each cluster over time. The accompanying table summarizes clustering characteristics, including cluster ID and label (LLR), average publication year, cluster size, silhouette score, and top 5 keywords for each cluster.

Subsequently, a clustering analysis of 592 keywords was performed using CiteSpace. The log-likelihood ratio (LLR) algorithm was applied to extract the nine largest clusters. As shown in **Figure 7A**, the clusters are labeled as follows: #0 Blood-Brain Barrier Disruption, #1 Angiogenesis, #2 Cerebral Blood Volume, #3 Vascular Endothelial Growth Factor, #4 Lipid Classes, #5 Enhanced Permeability, #6 Differential Permeability, #7 Type 1 Cannabinoid Receptor, and #8 Differential Diagnosis. The clustering network yielded a Q value of 0.3596, exceeding the 0.3 threshold, which indicates a meaningful modular structure. The average S value was 0.7212, also higher than the 0.7 benchmark, suggesting high homogeneity within clusters. In the figure, colored blocks represent different cluster regions, and the colored frames encompass the keywords within each cluster.

The temporal evolution of the nine clusters was further analyzed, as shown in **Figure 7B**. Cluster #0 Blood-Brain Barrier Disruption emerged as the core research theme in this field, positioned at the top of the figure. Its research trend has steadily increased since 1995 and peaked significantly around 2020, reflecting sustained scholarly attention and a deepening exploration of the BBB. In contrast, Cluster #3 Vascular Endothelial Growth Factor experienced a short-term surge between 1995 and 2000, followed by a rapid decline and subsequent stabilization at a relatively low level, indicating an early wave of interest in the relationship between VEGF and the BBB.

Other topics, such as Cluster #1 Angiogenesis and Cluster #2 Cerebral Blood Volume, displayed varied growth patterns across different periods, with considerable academic fluctuations and relatively stable activity in recent years. Clusters like #4 Lipid Classes, #5 Enhanced Permeability, and #7 Type 1 Cannabinoid Receptor began to attract attention around 2005, although their overall research intensity remains low, suggesting these areas are still in an exploratory phase. Notably, Cluster #6 Differential Permeability and Cluster #8 Differential Diagnosis exhibited a trajectory characterized by early emergence followed by subsequent decline. The accompanying table provides detailed information about the key clusters, reflecting the major research themes and the underlying knowledge structure in GBM and BBB research.

### 3.5 Co-citation analysis and evolution of references

**Figure 8:**
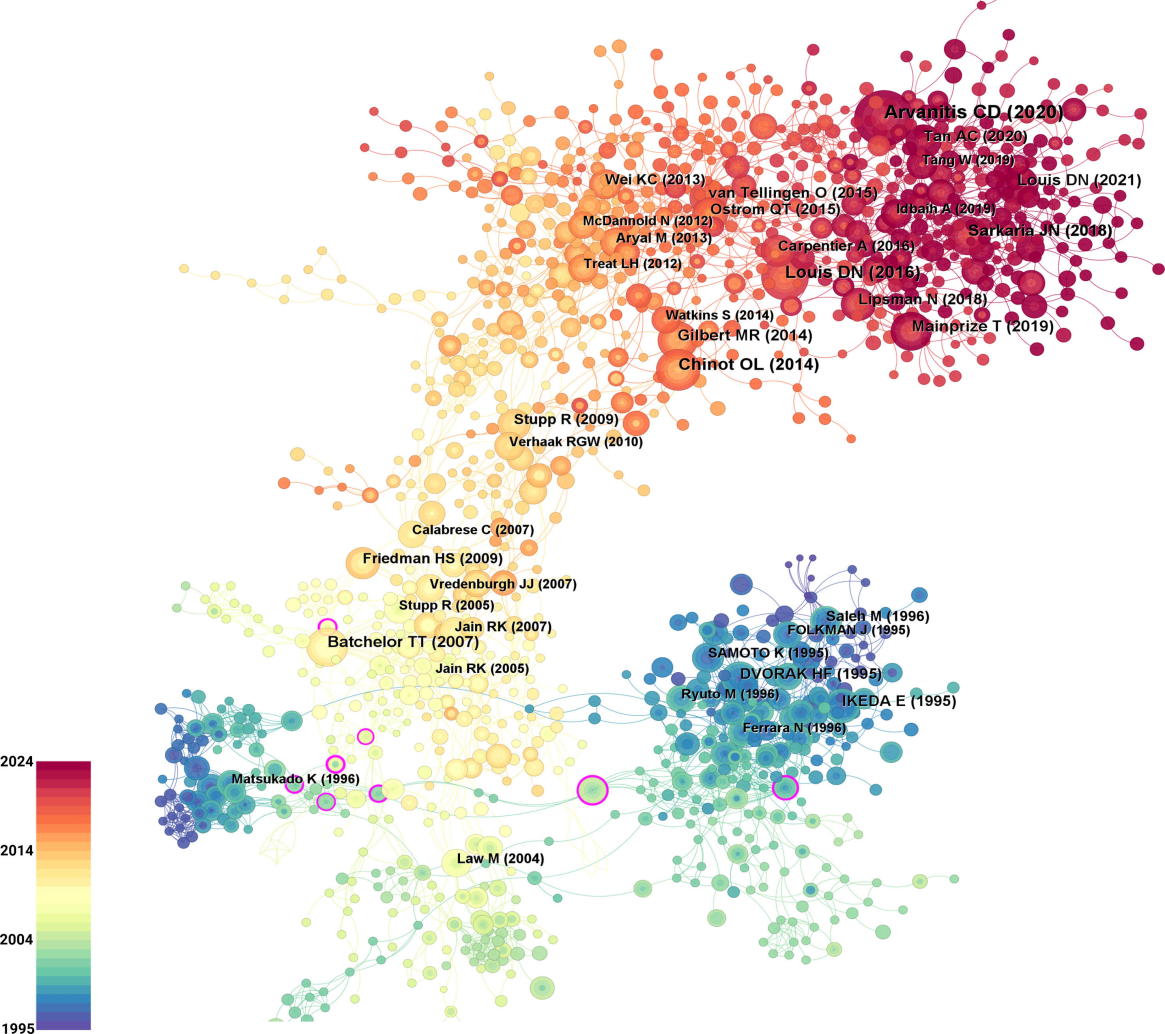
Co-citation analysis of references. Nodes represent individual references, with size indicating citation frequency and color intensity reflecting significance. Edges denote co-citation relationships between references.

A reference co-citation analysis was conducted in CiteSpace. Time slicing was set to 1995−2024 (1 year per slice). Node type: Reference. Link strength: cosine (default). Scope: Within slices (default). Selection criteria: g-index (k = 25 per slice). Pruning: Pathfinder, Pruning sliced networks, and Pruning the merged network were enabled. The merged network comprised 1,564 nodes and 3,476 links (network density = 0.0028). As shown in **Figure 8**, clusters are represented by different colors, illustrating the evolutionary paths of various research directions. Several highly cited works stand out within specific clusters, as indicated by larger node sizes representing citation frequency In the blue-green region: Folkman J (1995), Dvorak HF (1995), and Ferrara N (1995); in the yellow region: Batchelor TT (2007), Vredenburgh JJ (2007), and Jain RK (2007); in the orange region: Gilbert MR (2014), Wei KC (2014), and Chinot OL (2014); and in the red region: Sarkaria JN (2018), Arvanitis CD (2020), and Tan AC (2020). These publications form the intellectual backbone of the network and represent key contributions across different thematic domains. Further analysis of these references can help elucidate their respective research focuses and clarify the developmental trajectory of the field.

From the structure of the co-citation network, four major thematic areas can be identified, outlining the developmental trajectory of research on GBM and the BBB. Early foundational studies, represented by Folkman J (1995) and others in the blue-green cluster, established the critical role of VEGF in tumor angiogenesis. This was followed by research from Batchelor TT (2007) and others in the yellow cluster, which advanced anti-angiogenic therapeutic strategies, particularly the clinical application of bevacizumab. Publications such as Gilbert MR (2014), located at the interface between the foundational and translational phases (orange cluster), focused on optimizing BBB permeability and drug delivery pathways. More recent studies, exemplified by Sarkaria JN (2018) in the red cluster, have shifted attention toward molecular biomarkers, precision medicine, and combination therapies, indicating a transition in the field from mechanistic exploration toward clinical implementation.

**Figure 9:**
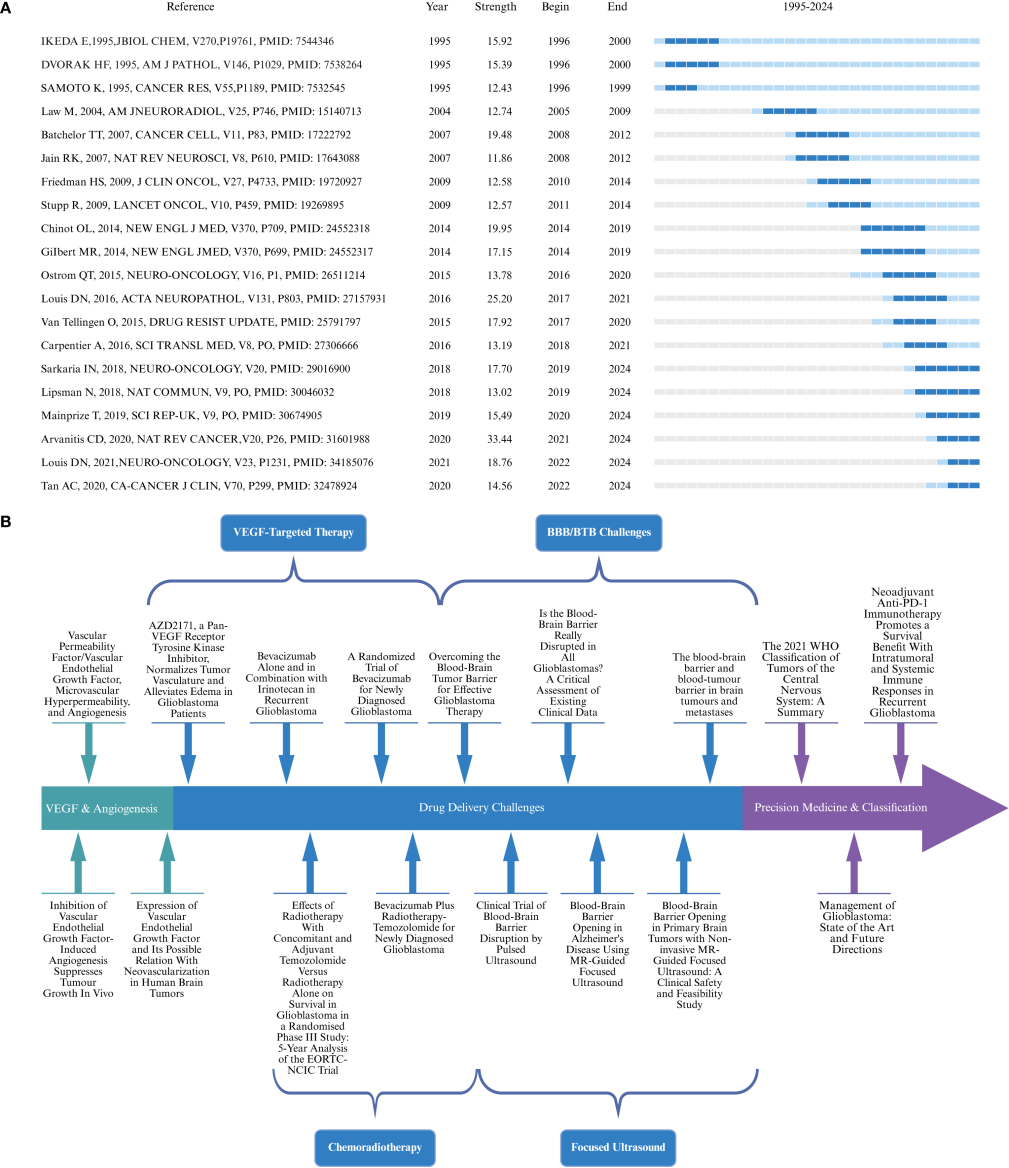
Citation burst strength and thematic evolution of influential references in GBM and BBB research. **(A)** Top 20 references with the strongest citation bursts. **(B)** Timeline of key studies illustrating the evolution of major research themes over time.

**Figure 9A** presents the top 20 most-cited references to date, highlighting their citation strength and burst duration. In terms of citation strength, the article by Arvanitis CD titled “The blood-brain barrier and blood-tumor barrier in brain tumors and metastases” demonstrated the highest citation intensity during the period from 2021 to 2024. Regarding burst end time, publications such as “Management of glioblastoma: State of the art and future directions” by Tan AC and “The 2021 WHO Classification of Tumors of the Central Nervous System: a summary” by Professor Louis DN showed strong citation bursts that continued through 2024. These influential works continue to serve as foundational references for future scholarship in the field.

Building upon the results of the co-citation analysis and citation burst detection, a developmental timeline of research on GBM and the BBB was constructed. As shown in **Figure 9B**, this figure systematically outlines the progression of key thematic stages, including VEGF & Angiogenesis, VEGF-Targeted Therapy, BBB/Blood-Tumor Barrier (BTB) Challenges, Chemoradiotherapy, FUS, and Precision Medicine & Classification. Representative landmark publications are listed for each phase, highlighting the evolutionary trajectory of the field and its major breakthroughs at the research frontier.

**Figure 10:**
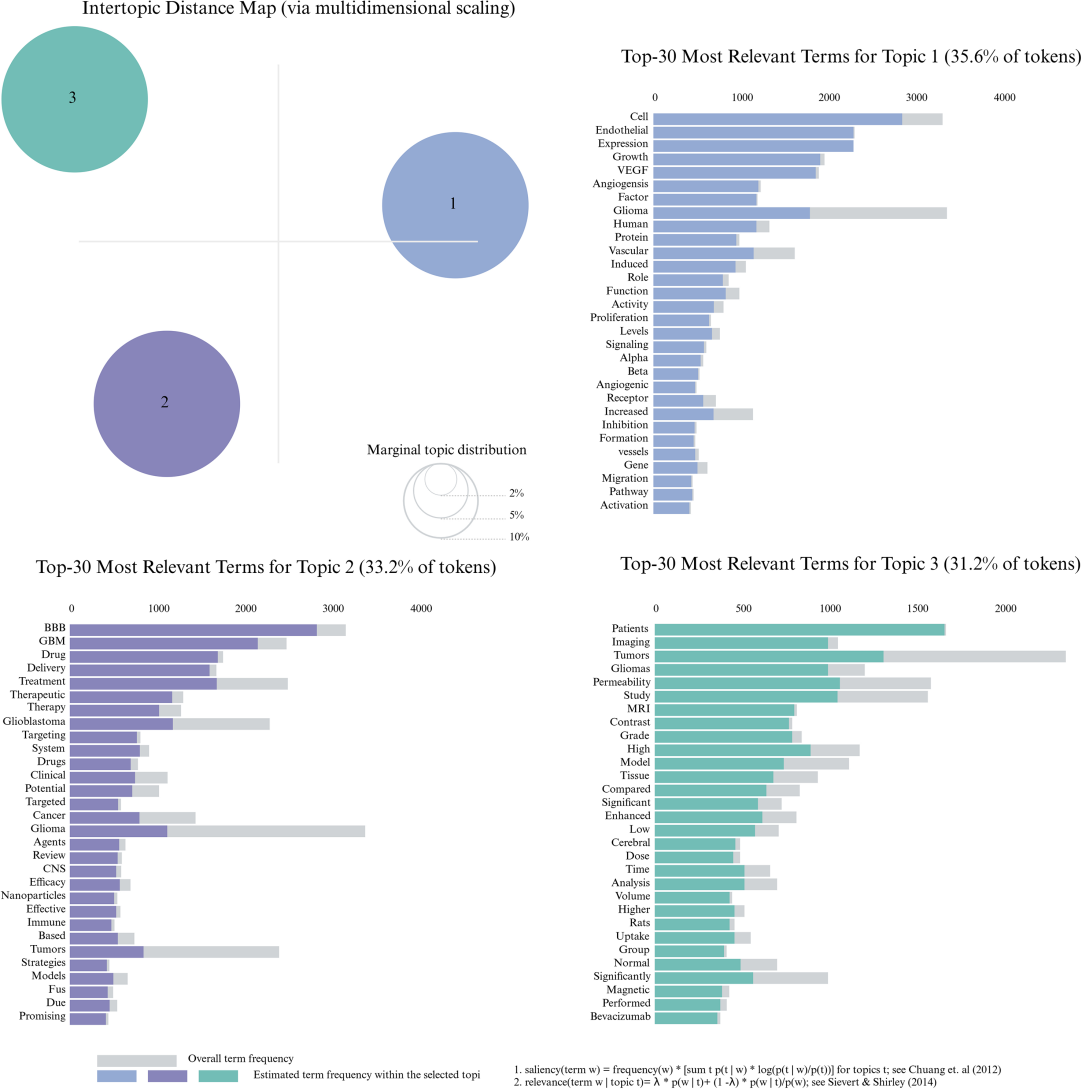
Topic modeling results based on LDA. Three major thematic clusters are identified. The intertopic distance map visualizes topic separation, and the top 30 most relevant terms for each topic are listed with their corresponding frequencies.

Based on the thematic content of the literature, text preprocessing was performed in R. Common stop words such as “background,” “objective,” “setting,” “methods,” and “results,” as well as punctuation, were removed. Additionally, low-frequency terms that appeared fewer than 10 times were excluded. The cleaned text was then converted into a DTM. On this basis, the number of topics was set to three, and LDA topic modeling was applied to identify latent themes beyond those identified through keyword clustering. As shown in **Figure 10**, the results were broadly categorized into three topics, with clearly defined boundaries and minimal overlap, indicating that research on GBM and the BBB has evolved into three relatively distinct yet complementary directions.

The first topic, Angiogenesis and Blood-Brain Barrier Disruption (accounting for approximately 35.6%), is characterized by keywords such as VEGF, angiogenesis, endothelial, expression, growth, glioma, vascular, induced, and inhibition. The second topic, Targeted Therapy and Drug Delivery in GBM (approximately 33.2%), includes keywords such as blood-brain barrier, delivery, therapy, glioblastoma, nanoparticles, agents, targeting, immune, and promising. The third topic, Imaging Assessment and Blood-Brain Barrier Permeability (approximately 31.2%), is associated with keywords such as permeability, MRI, contrast, patients, gliomas, cerebral, and volume.

Notably, the third topic highlights the detection and imaging of BBB status as an emerging thematic area. Common non-invasive imaging techniques, such as MRI and contrast-enhanced imaging, have been widely employed to assess changes in BBB permeability, drug distribution, and tumor hemodynamics. These imaging modalities not only facilitate effective evaluation of BBB integrity but also enable real-time monitoring of therapeutic responses, making them essential tools in clinical research. As a result, imaging-based BBB assessment has gradually developed into an independent research direction.

### 3.6 Dual-overlay analysis of research disciplines

**Figure 11:**
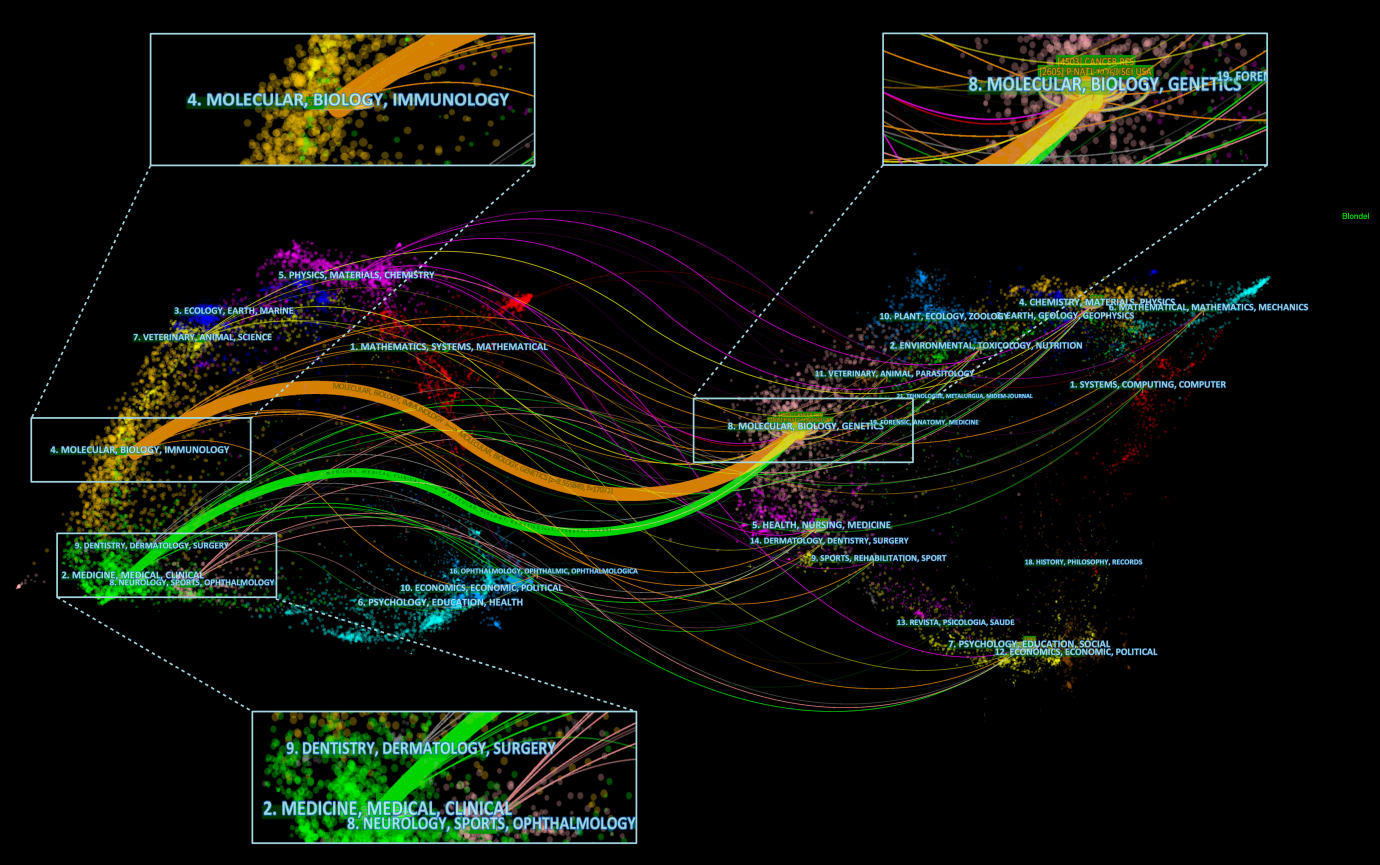
Dual-overlay map of research disciplines. Different colored clusters represent distinct research fields, and citation paths illustrate interdisciplinary connections over time.

In addition, a dual-map overlay analysis was conducted using CiteSpace to explore the disciplinary connections between citing and cited references. As shown in **Figure 11**, the citing literature is displayed on the left side of the map, while the cited literature appears on the right, providing a clear visualization of the primary knowledge flow and interdisciplinary linkages in the field of GBM and BBB research. Two major citation paths are highlighted in the map: the orange path originates from the field of Molecular Biology/Immunology, while the green path begins from Medicine/Medical/Clinical, Neurology/Sports/Ophthalmology, and Dentistry/Dermatology/Surgery. Both paths converge toward Molecular Biology/Genetics, indicating that, irrespective of whether the research originates from basic science or clinical medicine, recent studies increasingly converge on molecular-level mechanisms.

### 3.7 Topic and network patterns in PubMed clinical trials

Given that WoS data primarily emphasize basic research, we independently analyzed publications labeled as Clinical Trials in the PubMed database to further investigate trends in clinical research. The goal was to identify the core research focuses, intervention strategies, and the thematic evolution within this subset of literature.

**Figure 12:**
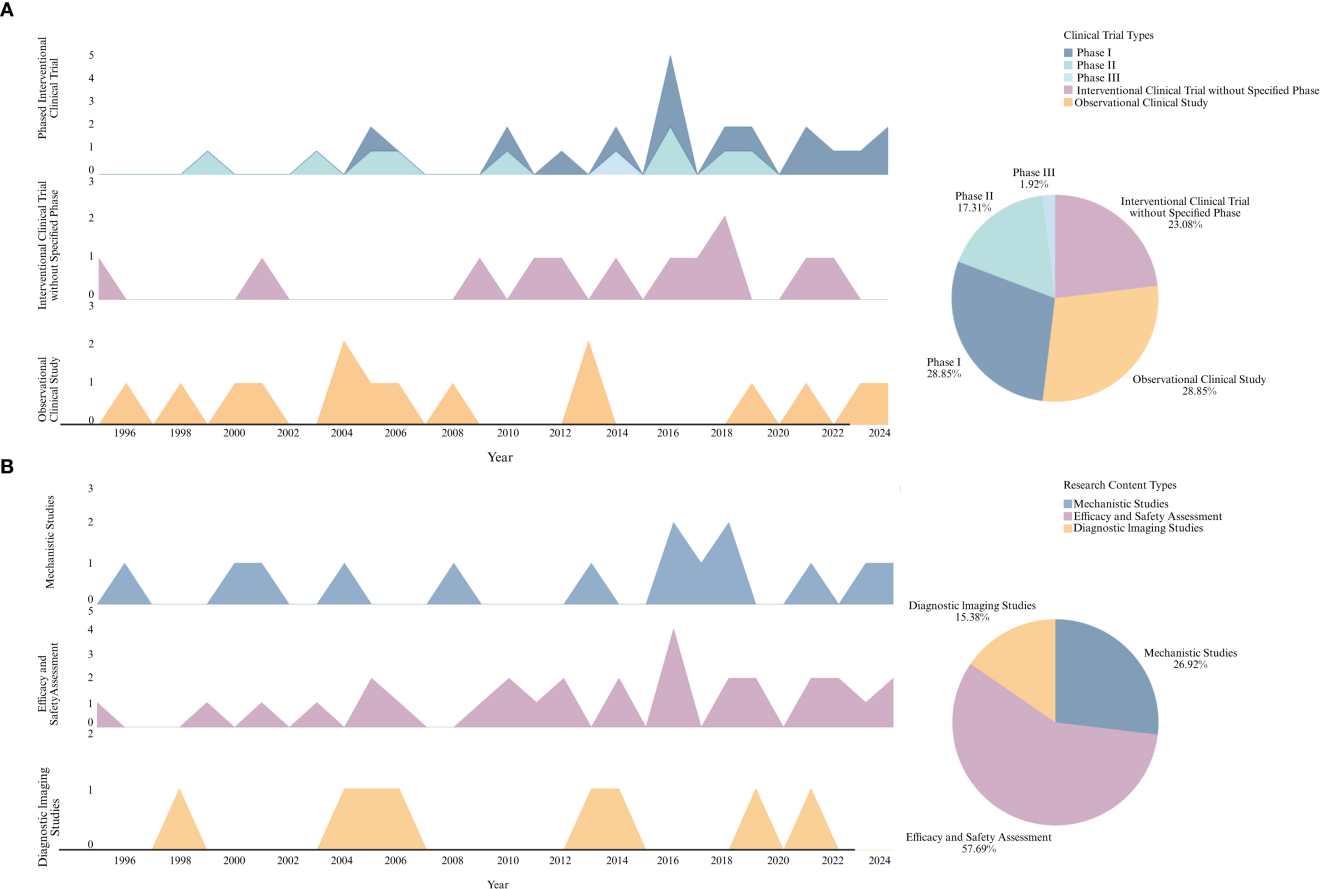
Clinical trial types and research content types in PubMed-identified studies. **(A)** Temporal distribution and composition of clinical trial types. **(B)** Temporal distribution and composition of research content types. The accompanying table lists representative keywords extracted from each research content type.

Using the R programming language, we categorized the included publications along two key dimensions: clinical trial type (Interventional Clinical Trial vs. Observational Clinical Study) and research content type (Mechanistic Studies, Efficacy and Safety Assessment, Diagnostic Imaging Studies). Corresponding faceted area plots and pie charts were generated. As shown in **Figure 12A**, Observational Clinical Studies constituted 28.85% of the total and were more prevalent in earlier years, whereas Interventional Clinical Trials accounted for 71.15% but were largely conducted after 2010. As shown in **Figure 12B**, Efficacy and Safety Assessment (57.69%) has remained the dominant focus, exhibiting a notable upward trend in recent years. Diagnostic Imaging Studies were limited in number and were active only sporadically across specific time periods. Subsequently, we performed text mining and keyword frequency analysis on each type of clinical trial to extract representative core research themes, which are listed in the accompanying table. Mechanistic Studies primarily focused on BBB permeability, VEGF, hypoxia, and P-gp. Efficacy and Safety Assessment studies emphasized themes such as chemotherapeutic agents, BBB disruption, combination therapy, and dose-response relationships. Diagnostic Imaging Studies mainly involved PET imaging, contrast-enhanced MRI, and perfusion imaging.

**Figure 13:**
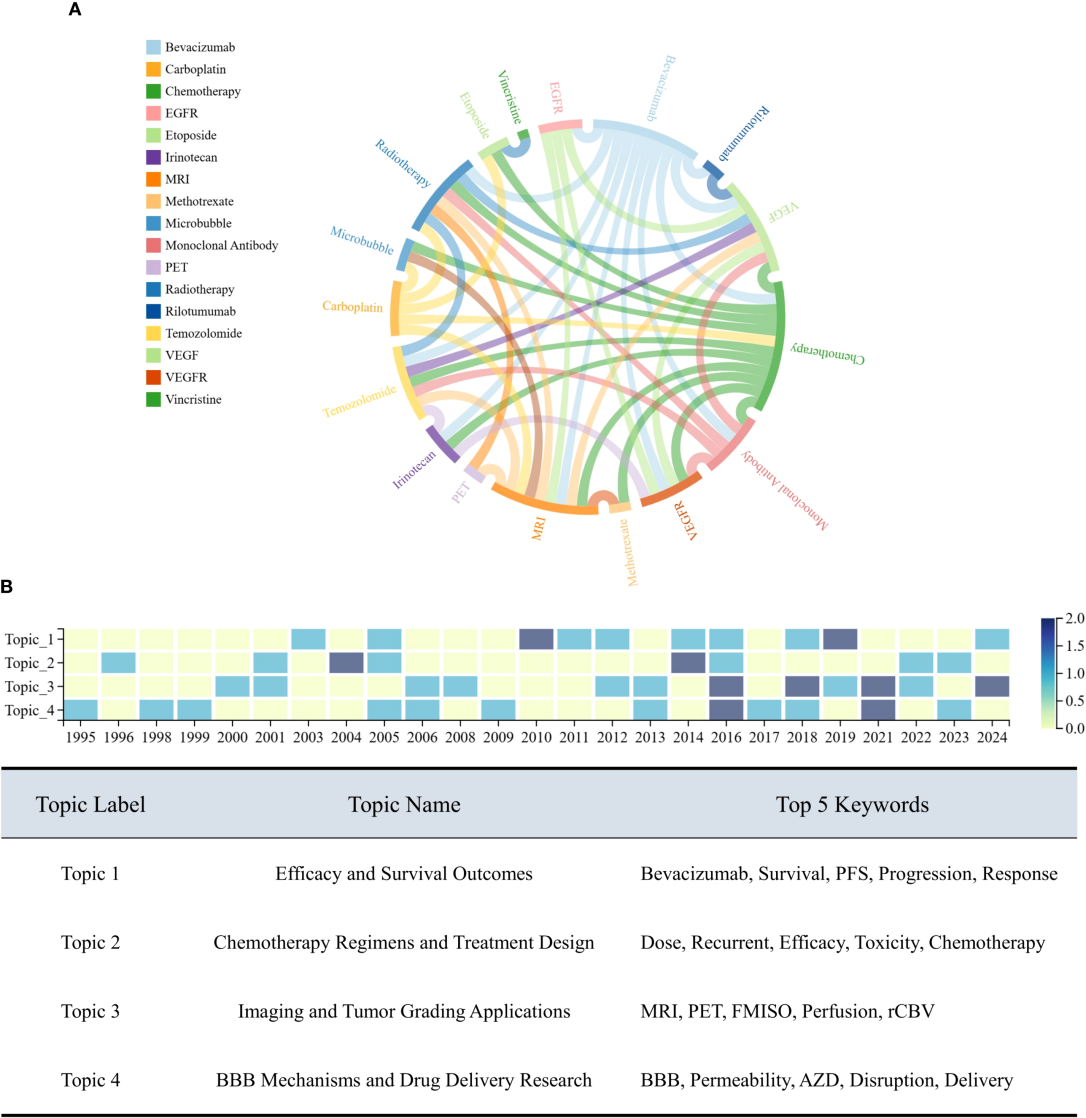
Intervention strategies and topic evolution in PubMed-identified clinical trials. **(A)** Co-occurrence network of intervention-related keywords. **(B)** Temporal evolution of latent research topics identified via LDA topic modeling. Heatmap illustrates the changing intensity of each topic from 1995 to 2024.The accompanying table lists the four major topics, their thematic labels, and the top five high-frequency keywords associated with each topic.

Building on this, we constructed a dictionary of intervention strategy-related keywords and matched them to the abstracts to analyze co-occurrence relationships. As illustrated in **Figure 13A**, Chemotherapy frequently co-occurred with a range of cytotoxic agents (e.g., Carboplatin, Vincristine, Etoposide), suggesting that multidrug combination therapy remains the mainstream approach. In contrast, nodes such as Monoclonal Antibody, EGFR, and VEGFR showed fewer connections, indicating that these strategies are still in exploratory phases and have not yet formed stable combinations with other interventions.

Furthermore, LDA topic modeling was applied to cluster the clinical trial literature into four latent research themes. A heatmap was constructed to visualize the temporal dynamics of each topic. As shown in **Figure 13B**, Topic 4 (BBB Mechanisms and Drug Delivery Research) has clearly emerged as a recent research hotspot, exhibiting a sharp rise after 2015 and intensified focus during 2020−2024. This trend reflects a shift in clinical trial priorities from conventional chemotherapy toward targeted therapy, advanced imaging, and novel drug delivery systems, aiming to achieve precision medicine. The top five high-frequency keywords for each topic are summarized in the accompanying table.

## 4 Discussion

Bibliometric analysis in the field of GBM and BBB research provides valuable insights into developmental trends and research hotspots, offering important guidance for future studies. In this study, we adopted a dual-database analysis strategy using both the Web of Science and PubMed databases to comprehensively capture both basic and clinical research outputs. In recent years, the increasing understanding of BBB structure and function, along with advances in the investigation of GBM invasion mechanisms, has emerged as a central focus of academic research. Our bibliometric analysis reveals that the United States ranks first in both the number of publications and total citations, highlighting its dominant position in this research domain. Most high-impact authors are affiliated with institutions in the United States and Europe. Although Chinese scholars have contributed a large number of publications, their average citation impact remains relatively low. Furthermore, extensive collaborative networks have formed among countries, institutions, and authors, with the United States, China, and several European nations playing central roles in global scientific cooperation.

Research in this field has evolved significantly over time. Early studies primarily focused on how GBM promotes its growth and spread by disrupting the BBB, particularly emphasizing the role of VEGF in tumor angiogenesis and increased BBB permeability. Subsequently, research shifted toward therapeutic strategies aimed at overcoming the BBB. This phase included the use of VEGF-targeted therapies such as bevacizumab, followed by the combination of chemotherapy and radiotherapy. More recently, emerging technologies like nanotechnology and FUS have been extensively investigated as innovative strategies to bypass the BBB. In the past few years, as our understanding of GBM−BBB interactions and the TME has deepened, two major research directions have emerged. First, studies increasingly leverage single-cell technologies and molecular profiling to investigate GBM cellular heterogeneity, aiming to enable precision medicine. Second, novel immunotherapeutic approaches, including chimeric antigen receptor (CAR) T-cell therapy and immune checkpoint inhibitors, have shown promising results in clinical trials. Whereas WoS-based analyses mainly revealed trends in tumor biology, molecular signaling, and therapeutic resistance, the PubMed-derived clinical trial corpus emphasized practical interventions, such as multimodal drug delivery and imaging-based treatment evaluation. This complementary dual-perspective approach enabled a more integrated understanding of how fundamental discoveries interface with clinical innovation in the GBM−BBB field.

### 4.1 Global publication trends and national impact analysis

From 1995 to 2024, research in the field of GBM and the BBB has demonstrated a continuous upward trend, with an explosive increase in annual publication output observed after 2015. This pattern reflects growing scholarly interest in GBM, where the tumor’s location and poor prognosis have made BBB research a central and unavoidable issue. In light of this trend, countries such as the United States, China, Germany, the United Kingdom, and Canada are at the forefront of research in this area. This leadership is largely attributed to their advantages in research resources, infrastructure, technology, and funding, enabling them to conduct both high-quality basic and clinical research, which in turn drives academic productivity and citation impact. That said, despite China’s notable performance in terms of total publications and cumulative citations, its average citations per article remain relatively low. This suggests that the overall research impact of Chinese publications has yet to attain the international recognition seen in the United States and other Western countries. To enhance its academic influence, China must focus on improving research quality, fostering deeper international collaboration, and increasing the global visibility and impact of its contributions in this field.

### 4.2 Core authors and high-impact journals analysis

According to Price’s Law, the threshold for identifying core authors is calculated as $M=0.749\times \sqrt{N_{max}}$ where Nmax represents the maximum number of publications by a single author within a given period. Among the top 20 authors, Xue, Y. ranked first with 53 publications, resulting in an M value of 5.45 according to Price’s Law. In practice, to ensure inclusivity and account for the discrete nature of publication counts, authors with three or more publications were considered core contributors in this analysis. Based on this threshold, in the GBM and BBB research field in China, a total of 332 core authors were identified, accounting for 49.55% of all contributing authors (670 individuals), and producing 1,619 articles, which represent 48.58% of the total publications (3,332 articles). This proportion, which approaches 50%, suggests that a relatively stable core author group has formed in China. However, no author has yet entered the category of high-impact researchers, indicating that there is still room for improvement in terms of research quality. High-impact journals in this field are characterized by rigorous academic standards and wide international recognition. Researchers tend to publish high-quality work in these journals, which have a significant influence on the direction of the field. Therefore, in addition to tracking highly cited publications, it is equally important to monitor recent research published in these core journals, particularly studies that drive innovation and advance the understanding of the relationship between GBM and the BBB.

### 4.3 Institutional and author collaboration network analysis

Although leading Chinese institutions have strong domestic footprints, they continue to face headwinds in international collaboration and global impact. For example, the National Cancer Institute (NCI), which has a domestic influence comparable to Fudan University, has a TLS of 65, more than twice that of Fudan (TLS = 28). Even Harvard Medical School (Citations: 3,169; TLS = 65) exceeds Fudan substantially in network connectivity. This pattern indicates that China’s top institutions are highly active domestically but are less embedded in the global co-authorship network, an integration gap that helps explain lower average citation impact. Beyond network structure, the composition of research output also matters. Although China accounts for about 23.0% of global basic-research output, its share of clinical-trial publications in this field is only 9.4%, pointing to weaker translational/clinical impact relative to volume. In addition, many publications are single-center or retrospective, with limited participation in large multicenter clinical trials, which further reduces international visibility and citations. Together, these findings suggest that improving impact will require: 1) deepening cross-border collaboration with high-impact partners to raise TLS and co-authorship diversity; 2) rebalancing the portfolio toward rigorously designed basic and translational studies; and 3) greater participation in multicenter, prospective clinical trials. These steps would help narrow the gap between domestic prominence and global academic influence. At the author level, Patrick Y. Wen stands out as a key collaborator in the field, as evidenced by the dense connectivity of his co-authorship network. Specifically, a review of his recent publications highlights his significant contributions to clinical trials and therapeutic studies on GBM (12–14). Furthermore, he participated in the revision of response assessment criteria for GBM, such as the revised Response Assessment in Neuro-Oncology (RANO) criteria (15). Wen has authored several highly cited review articles, including “*Exciting New Advances in Neuro-Oncology: The Avenue to a Cure for Malignant Glioma*,” “*Toward Precision Medicine in Glioblastoma: The Promise and the Challenges*,” and “*Immunotherapy Advances for Glioblastoma*.” Taken together, these contributions highlight the team’s sustained commitment to personalized precision medicine and immunotherapy and have garnered significant academic prestige. In contrast, while Chinese scholars like Sun Tao and Liu Jun are gradually gaining influence, they still remain at the periphery of the collaboration network, indicating considerable room for academic advancement. Moving forward, they may benefit from adopting the collaborative models of teams led by Patrick Y. Wen or Reardon David A, by actively participating in international research initiatives and expanding cross-team partnerships to further elevate the global impact of their scientific contributions.

### 4.4 Analysis of research themes and hotspot evolution

Based on keyword co-occurrence and clustering analysis, it is clear that GBM and BBB are the central keywords within this research domain. The majority of current studies focus on exploring the complex interplay between GBM and the BBB.

Among these themes, particularly VEGF and its impact on BBB permeability emerged as a focal point in early investigations. A review of literature from this period suggests that the expression of VEGF was closely associated with tumor vascularization, cerebral edema, and necrosis, which are key hallmarks of GBM progression. Tumor cells were found to secrete VEGF to disrupt the BBB, thereby increasing vascular permeability and creating a favorable microenvironment for tumor growth. Additionally, the pathogenesis of GBM involves complex genetic alterations and chromosomal abnormalities, including p53 mutations and the overexpression of EGFR and VEGF. These molecular mechanisms contribute to tumor progression, invasiveness, and therapeutic resistance (16–18). As VEGF research advanced, it became evident that VEGF is highly expressed around necrotic tumor regions and plays a role in hypoxia-induced angiogenesis. Moreover, other growth factors such as Epidermal Growth Factor (EGF) and Platelet-Derived Growth Factor (PDGF) were also implicated in the malignant progression of gliomas (19, 20).

Building on these mechanistic insights, researchers began to explore therapeutic interventions targeting VEGF signaling. As a result, targeted therapies against VEGF, particularly those involving clinical trials with bevacizumab, were subsequently initiated. Studies demonstrated that bevacizumab prolonged progression-free survival (PFS) (10.7 months vs. 7.3 months); however, there was no significant difference in overall survival (OS) between the bevacizumab group (15.7 months) and the placebo group (16.1 months), indicating that the drug delays disease progression without improving overall survival (21, 22). Moreover, treatment strategies relying solely on VEGF inhibition in GBM are limited, as they may not adequately address distant tumor spread or other pathological factors (23). Specifically, GBM exhibits high heterogeneity, with different tumor regions responding differently to VEGF inhibition. Additionally, single-agent administration via conventional routes leads to uneven drug distribution, which may induce tumor vascular instability and cause local tumor hypoxia and insufficient blood perfusion. Hypoxic conditions activate the production of hypoxia-inducible factor-1α (HIF-1α) (24), a process that may further promote immune evasion in the tumor microenvironment, such as upregulated expression of programmed death-1 (PD-1)/programmed death-ligand 1 (PD-L1) and cytotoxic T-lymphocyte-associated protein 4 (CTLA-4), as well as the production of growth factors like fibroblast growth factor and transforming growth factor beta (TGF-β). This vascular instability not only enhances tumor invasiveness but also facilitates tumor escape through vascular remodeling driven by factors like angiopoietin-2 (Ang-2) (25). Furthermore, Yuji Piao and Ameratunga M et al. emphasized the significant limitations of using VEGF inhibitors as monotherapy for GBM. While anti-VEGF therapy can delay tumor growth, GBM often rapidly develops resistance. In resistant tumors, the expression of stem cell-related markers such as Nestin and Sox2 is significantly elevated. Additionally, tumors evade treatment through increased infiltration of myeloid immune cells, particularly CD11b^+^ cells, and by undergoing epithelial-mesenchymal transition (EMT). These findings suggest that single-agent VEGF-targeted therapy fails to address the high heterogeneity and resistance of GBM. Therefore, treatment strategies must be diversified, and combining immune therapies or other targeted treatments may provide more effective therapeutic options (26, 27).

Given the limitations of anti-VEGF monotherapies, a range of strategies have emerged to enhance BBB permeability and improve therapeutic efficacy, including nanoparticle-based delivery systems and ultrasound-mediated BBB disruption. These approaches have been shown not only to significantly improve drug delivery efficiency and reduce off-target toxicity to normal brain tissue, but also to enhance drug bioavailability and targeting specificity, demonstrating substantial clinical potential (28–31). Joelle P. Straehla’s team has developed functionalized nanoparticles (AP2-NPs) to encapsulate cisplatin (CDDP), enabling targeted delivery across the BBB via low-density lipoprotein receptor-related protein 1 (LRP1)-mediated transcytosis. This results in higher intratumoral accumulation and improved therapeutic efficacy of CDDP (32). Additionally, nanoparticles loaded with RNA interference-based spherical nucleic acids (SNAs) have shown promising results in a phase 0 clinical trial for recurrent GBM (rGBM). As an adjunctive tool for chemotherapy, FUS has also been assessed for its ability to transiently disrupt the BBB in rGBM patients. Ko-Ting Chen and colleagues conducted a series of phase I clinical trials demonstrating that controlled BBB opening can be safely achieved at acoustic pressures ≤ 0.68 mechanical index (MI), while higher intensities (0.81 MI) may induce immune activation within the TME (33).

**Figure 14:**
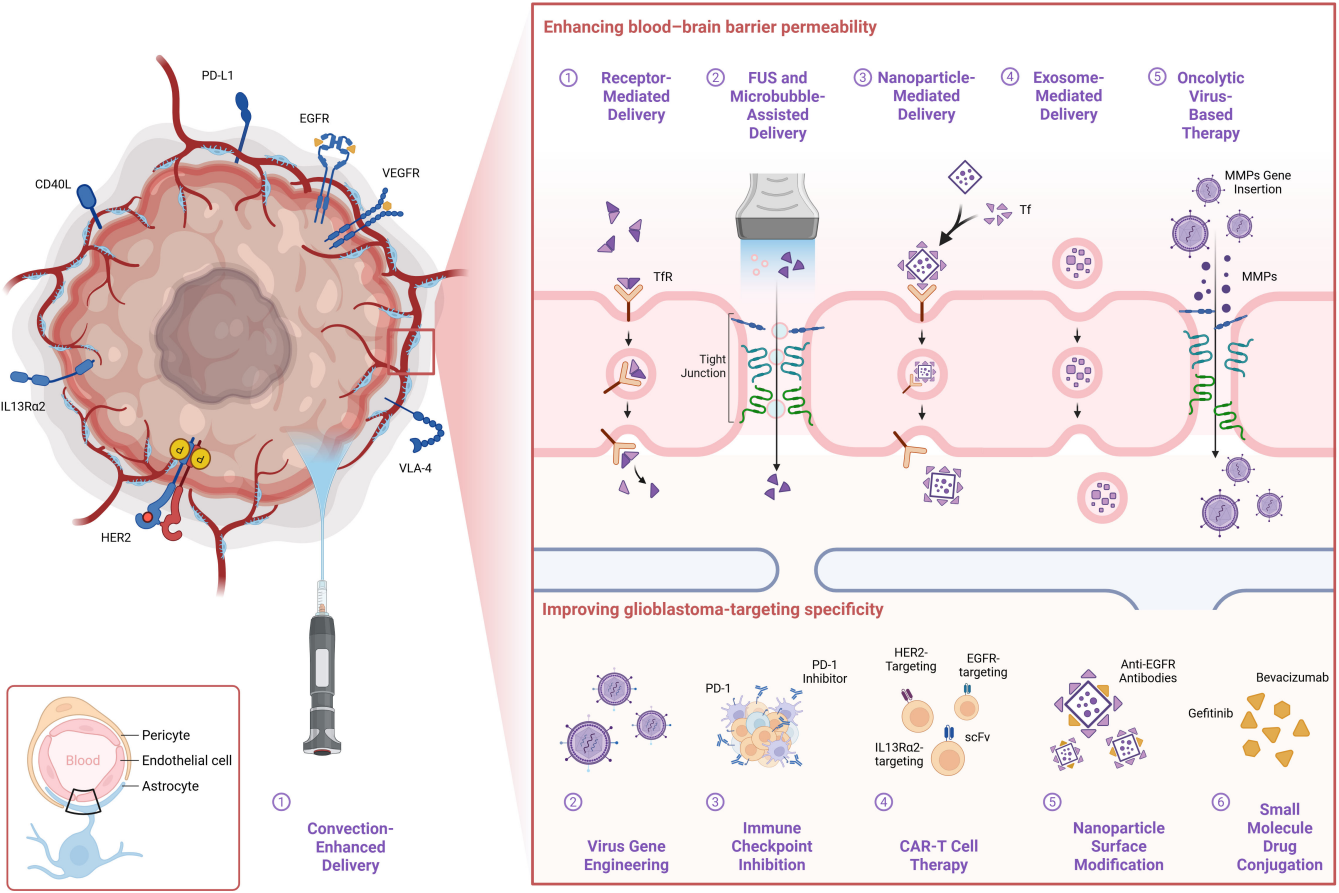
Schematic illustration of therapeutic strategies targeting GBM and the BBB. This figure presents various approaches currently under investigation to enhance BBB permeability and improve glioblastoma-targeting efficiency.

At the same time, in parallel with these physical and nanotechnological approaches, immunotherapy has emerged as a complementary and increasingly promising strategy. These include immune checkpoint inhibitors, CAR T-cell therapy (34), and oncolytic viruses, which aim to improve treatment specificity and enhance BBB permeability, thereby advancing the goals of precision medicine. Specifically, immune checkpoint inhibitors, including CTLA-4 and PD-1/PD-L1 blockers, have demonstrated therapeutic potential in glioblastoma (GBM), as affirmed by multiple studies (35–37). In 2020, Professor David A. Reardon’s team conducted the first phase III randomized controlled trial to assess the efficacy of the PD-1 inhibitor nivolumab in rGBM patients. Although the overall clinical outcome was unsatisfactory, the subgroup of patients without corticosteroid use and with methylated O6-methylguanine-DNA methyltransferase (MGMT) status showed increased sensitivity to nivolumab, suggesting that these factors may serve as potential predictive biomarkers for immunotherapy efficacy (38). Due to the high heterogeneity of GBM, antigen escape is common, posing a major challenge to immunotherapy. Consequently, extensive research has focused on antigens that are either GBM-specific or highly expressed in GBM, such as interleukin-13 receptor subunit alpha-2 (IL13Rα2), epidermal growth factor receptor variant III (EGFRvIII), and human epidermal growth factor receptor 2 (HER2) (39). To overcome the limitations of single-target approaches, recent efforts have shifted toward multi-targeted approaches, localized delivery, and TME modulation (40–42). Compared with immune checkpoint inhibitor therapies, CAR T-cell therapy has shown more promising clinical outcomes (43, 44), For example, in a clinical trial targeting IL13Rα2, disease stabilization was achieved in 50% of patients with rGBM, including two cases of partial response and two cases of complete response, with response durations of 7.5 months and over 66 months, respectively (45) research on oncolytic viruses primarily focuses on three main strategies: (1) genetically modifying viral capsid proteins to enable penetration of the BBB (46); (2) bypassing the BBB through direct intratumoral injection (47); (3) engineering oncolytic viruses to express immunomodulatory genes, such as interleukin-12 (IL-12), interleukin-15 (IL-15), TNF-related apoptosis-inducing ligand (TRAIL), and anti-PD-1, to enhance antitumor immune responses (48), Nature published the first phase I clinical trial of CAN-3110, an engineered oncolytic herpes simplex virus type 1 (HSV-1), in rGBM patients. This trial evaluated the safety and preliminary efficacy of CAN-3110, providing strong clinical evidence for oncolytic virotherapy in rGBM (49) shown in **Figure 14**, the main therapeutic strategies currently employed to overcome BBB limitations and enhance GBM targeting are illustrated, highlighting recent advances and future directions in this field.

While treatment strategies have diversified, diagnostic and monitoring approaches have also advanced. Among these, imaging technologies such as MRI and magnetic resonance spectroscopy (MRS) play a crucial role in guiding therapy and distinguishing GBM from other types of brain tumors (50). As a result, the differential diagnosis of GBM has progressed toward a more precise and multidimensional approach. By analyzing the ratios of tumor metabolites, researchers have enhanced the accuracy in differentiating tumor types (51). Additionally, molecular characteristics of GBM, such as isocitrate dehydrogenase (IDH) and BRAF V600E mutations, are now included in diagnostic and prognostic assessments. The identification of these genetic alterations not only enhances the precision of tumor classification but also provides a potential basis for targeted therapy (52, 53). Moreover, imaging technologies are also employed to distinguish between pseudoprogression and true tumor progression, thereby assisting clinicians in evaluating post-treatment responses (54).

As indicated by the clustering results, research interest in the fields of differential permeability and differential diagnosis has declined in recent years. (1) Differential Permeability: Earlier studies primarily focused on describing the differences in BBB permeability between the core and peripheral regions of GBM. However, with advancements in imaging techniques and molecular biology, research has increasingly shifted toward microscopic mechanisms. These include investigations of tight junction proteins, ATP-binding cassette (ABC) transporters, and dysregulated molecular pathways such as the Wnt/β-catenin signaling pathway (55–57). As a result, macro-level studies on permeability differences have gradually lost momentum. (2) Differential Diagnosis: In the early stages, imaging modalities such as MRI, CT, and PET played a central role in distinguishing GBM from other intracranial pathologies, resulting in a surge of related studies. However, the rise of molecular pathological diagnostics, including IDH mutations, 1p/19q codeletion, and O6-methylguanine-DNA methyltransferase (MGMT) promoter methylation, has redefined the diagnostic and classification standards for gliomas (58, 59). Consequently, the role of imaging-based differential diagnosis has been gradually marginalized. Furthermore, the increasing convergence of imaging techniques and their inherent limitations in diagnostic accuracy have further reduced the clinical value and research attractiveness of traditional imaging-based approaches.

### 4.5 Milestone literature analysis

An analysis of six highly cited publications up to 2024 reveals that the relationship between GBM and the BBB has remained a central and continuously evolving research focus. (1) Sarkaria IN (2018, Neuro-Oncology): This study highlighted that although the BBB is disrupted in certain regions of GBM, some areas retain an intact barrier. These intact regions may represent critical sites of therapeutic failure, underscoring the need for future treatment strategies to specifically target these protected zones in order to enhance therapeutic efficacy. (2) Lipsman N (2018, Nature Communications) and Mainprize T (2019, Scientific Reports-UK): These studies focused on the use of magnetic resonance-guided focused ultrasound (MRgFUS) to noninvasively open the BBB. Both articles recognized the safety profile of MRgFUS and its promising potential for clinical applications in enhancing drug delivery to brain tumors. (3) Arvanitis CD (2020, Nature Reviews Cancer): This review expanded the scope of BBB-related research by exploring the interplay between the BBB and the BTB and their combined influence on drug delivery. The study emphasized that, although vascular heterogeneity and the dynamic nature of the BBB present significant therapeutic challenges, modulating BTB transcriptional programs may improve intratumoral drug distribution and increase drug concentrations within tumor regions, thereby enhancing therapeutic outcomes. (4) Louis DN (2021, Neuro-Oncology): This article summarizes updates in the fifth edition of the World Health Organization Classification of Tumors of the Central Nervous System (WHO CNS5). It emphasized the central role of molecular diagnostics in tumor classification and highlighted key molecular markers that facilitate accurate clinical diagnosis and personalized treatment of gliomas. (5) Tan AC (2020, CA: A Cancer Journal for Clinicians): This comprehensive review outlined the current standard-of-care approaches for GBM, including surgery, radiotherapy, and chemotherapy, and explored the future potential of immunotherapy and precision oncology. The study noted that, despite progress with existing therapies, the BBB and TME continue to pose major therapeutic challenges. It underscored the urgent need to develop more effective treatment strategies.

Taken together, these landmark studies highlight the complexity of the BBB and TME in the context of GBM therapy. They collectively emphasize that integrating advanced technologies, such as MRgFUS and precision oncology, may enable the development of more targeted and effective therapeutic approaches, offering promising directions for future treatment breakthroughs.

While this study provides a comprehensive bibliometric analysis of GBM and the BBB, it has several limitations. First, because of indexing delays in WoS and PubMed, very recent 2024 publications may be underrepresented. To preserve cross-database comparability, we did not integrate preprints; accordingly, some emerging evidence available on preprint servers (e.g., medRxiv) may not be captured, and future work could consider analyzing such sources separately. Second, although we employed a purposive and standardized search strategy (including MeSH-informed terms and manual curation), reliance on specified keywords may have led to the omission of studies using alternative terminology, thereby affecting completeness. Lastly, we did not disaggregate the contributions of affiliated institutions or assess the academic influence of individual investigators within them, which limits inferences about institutional dynamics and patterns of knowledge dissemination.

## 5 Conclusion

Through a bibliometric analysis, this study summarizes the research trends in the field of GBM and the BBB, offering valuable insights for future investigations. Earlier studies primarily focused on how GBM disrupts the BBB to facilitate tumor growth and invasion. In recent years, however, research priorities have gradually shifted toward angiogenesis, optimization of drug delivery, and the development of precision medicine. With growing insights into TME and cellular heterogeneity, emerging therapeutic strategies such as immunotherapy, nanotechnology, and focused ultrasound have become prominent hotspots and are progressively advancing into clinical trials, accelerating the translation of basic research findings. By integrating perspectives from both basic and clinical research, this study presents a comprehensive picture of the multidimensional development of GBM−BBB research and offers a systematic reference for the coordinated advancement of mechanistic studies and translational applications.

## Data Availability

Publicly available datasets were analyzed in this study. This data can be found here: The datasets analyzed in this study were obtained from publicly accessible sources, including the Web of Science (https://www.webofscience.com/) and PubMed (https://pubmed.ncbi.nlm.nih.gov/). Search strategies and inclusion criteria are detailed in the Methods section.
