# Supplementary material for: Scientometric analysis of glioblastoma and blood-brain barrier research (1995−2024): evolving trends and therapeutic challenges
Source: Front Oncol. 2025 Sep 25;15:1649414. doi: 10.3389/fonc.2025.1649414 (PMC12507556; doi:10.3389/fonc.2025.1649414)
Supplement: Supplementary file 1 [file DataSheet1.pdf]

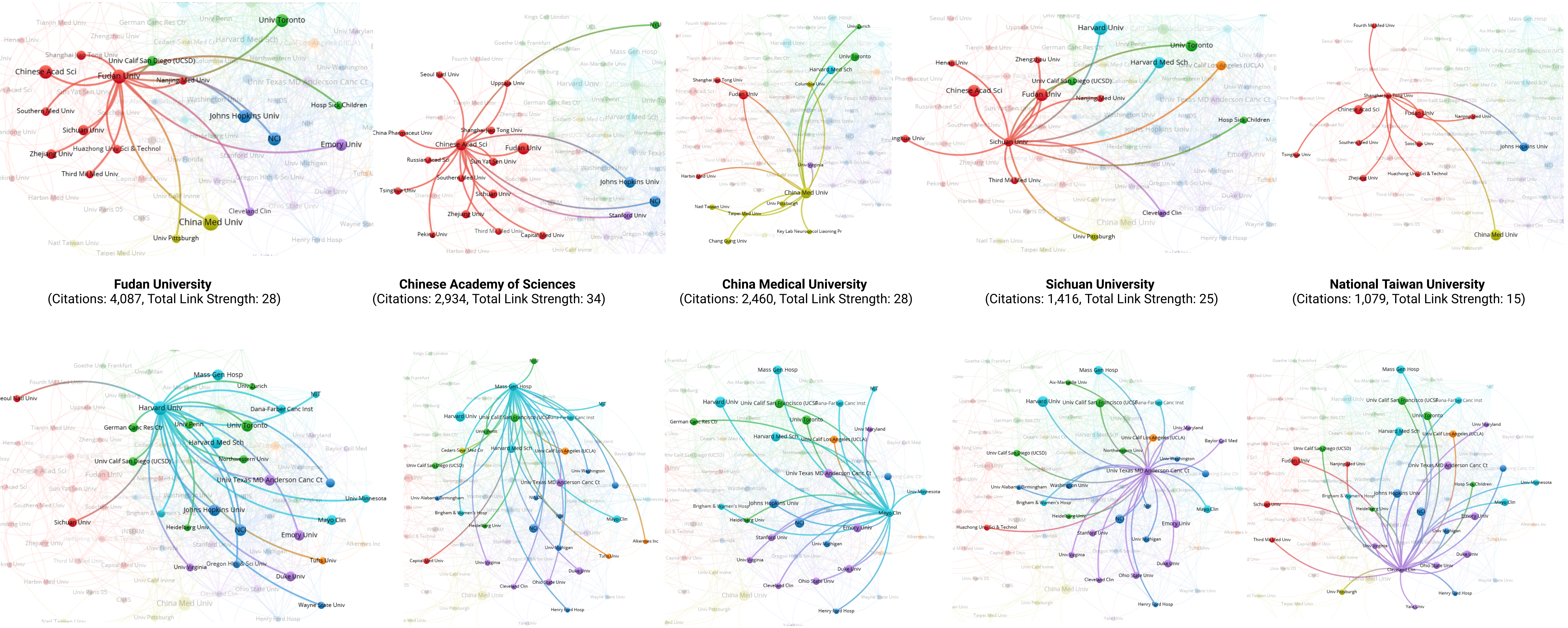

Fudan University
(Citations: 4,087, Total Link Strength: 28)

Chinese Academy of Sciences
(Citations: 2,934, Total Link Strength: 34)

China Medical University
(Citations: 2,460, Total Link Strength: 28)

Sichuan University
(Citations: 1,416, Total Link Strength: 25)

National Taiwan University
(Citations: 1,079, Total Link Strength: 15)

Harvard University
(Citations: 13,564, Total Link Strength: 80)

Massachusetts General Hospital
(Citations: 10,728, Total Link Strength: 80)

Mayo Clinic
(Citations: 8,479, Total Link Strength: 50)

University of Texas MD Anderson Cancer Center
(Citations: 7,798, Total Link Strength: 65)

Cleveland Clinic
(Citations: 6,641, Total Link Strength: 30)

Supplementary Figure S1. Comparison of Top 5 Institutions by Citations Global and China
