# Supplementary figures and images for: Scientometric analysis of glioblastoma and blood-brain barrier research (1995−2024): evolving trends and therapeutic challenges
Source: Front Oncol. 2025 Sep 25;15:1649414. doi: 10.3389/fonc.2025.1649414 (PMC12507556; doi:10.3389/fonc.2025.1649414)

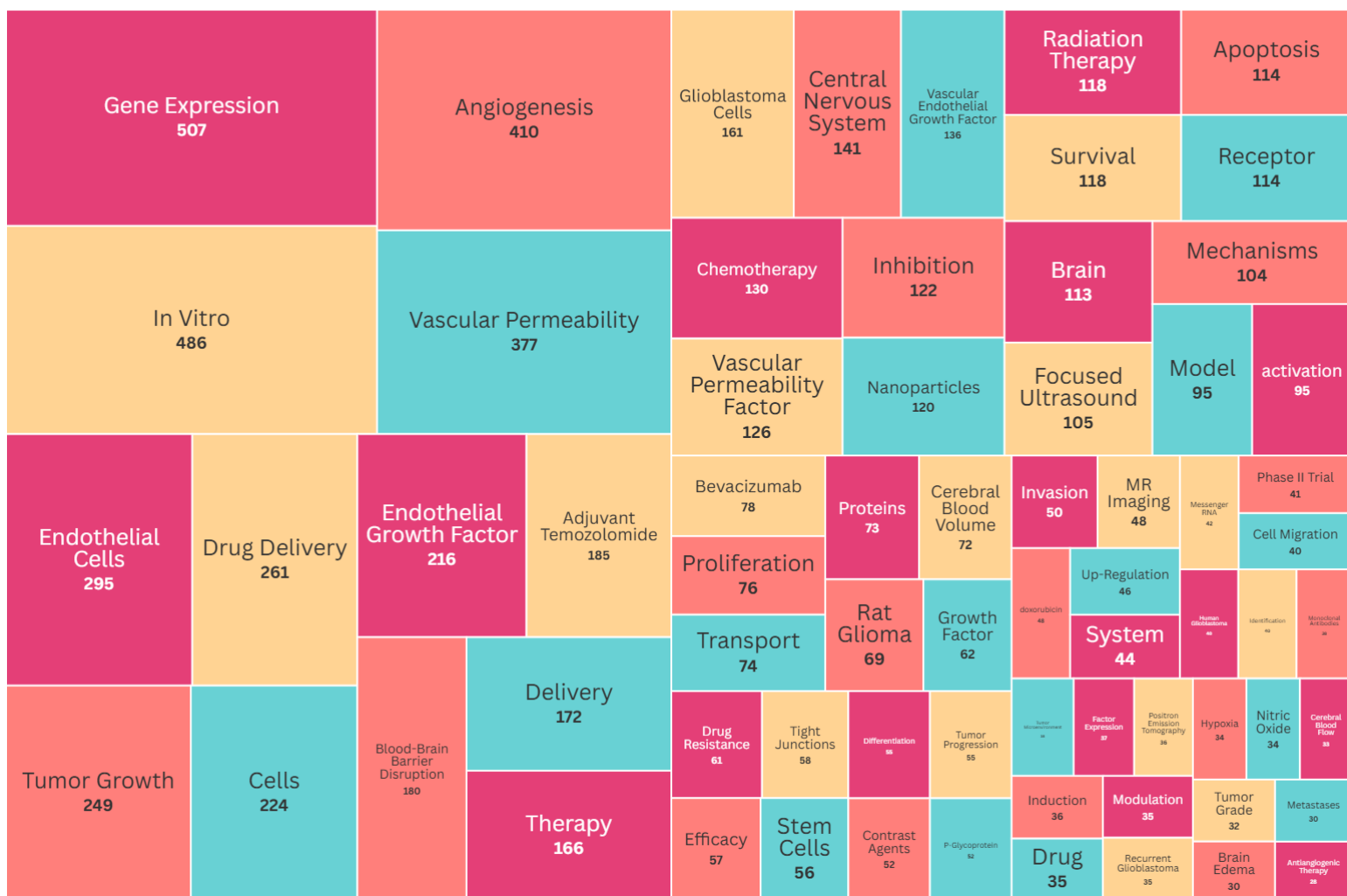

### Supplementary Figure S2. Keyword Co-occurrence Analysis

Supplement: Supplementary file 2 [file DataSheet2.pdf]
