# Supplementary material for: Scientometric analysis of glioblastoma and blood-brain barrier research (1995−2024): evolving trends and therapeutic challenges
Source: Front Oncol. 2025 Sep 25;15:1649414. doi: 10.3389/fonc.2025.1649414 (PMC12507556; doi:10.3389/fonc.2025.1649414)
Supplement: Supplementary file 3 [file DataSheet3.pdf]

Database	Search Query	Article Types
Web of Science	TS=(glioma* OR glioblastoma*) AND TS=("blood-brain barrier") AND TS=(structur* OR function* OR remodel* OR disrupt* OR impair* OR alter*)	Articles and Reviews
PubMed	("glioma"[Title/Abstract] OR "glioblastoma"[Title/Abstract] OR "gliomas"[Title/Abstract]) AND ("blood-brain barrier"[MeSH Terms] OR "blood-brain barrier"[Title/Abstract] OR "BBB"[Title/Abstract] OR "vascular permeability"[MeSH Terms] OR "vascular endothelial"[Title/Abstract] OR "endothelial cell"[Title/Abstract] OR "barrier function"[Title/Abstract] OR "barrier structure"[Title/Abstract]) AND ("structure"[Title/Abstract] OR "function"[Title/Abstract] OR "remodeling"[Title/Abstract] OR "dysfunction"[Title/Abstract] OR "integrity"[Title/Abstract] OR "permeability"[Title/Abstract] OR "disruption"[Title/Abstract] OR "alteration"[Title/Abstract] OR "modulation"[Title/Abstract])	Clinical Trials

Supplementary Table S1. Database search strategies and article-type filters for WoS and PubMed.
