# Supplementary material for: Scientometric analysis of glioblastoma and blood-brain barrier research (1995−2024): evolving trends and therapeutic challenges
Source: Front Oncol. 2025 Sep 25;15:1649414. doi: 10.3389/fonc.2025.1649414 (PMC12507556; doi:10.3389/fonc.2025.1649414)
Supplement: Supplementary file 4 [file DataSheet4.pdf]

Frequency	Original Term	Standardized Term
2	acid	Acid Amide Hydrolase
2	acid amide hydrolase	Acid Amide Hydrolase
93	activation	Activation
18	adjuvant temozolomide	Adjuvant Temozolomide
1	albino-rat	Albino Rat
8	analog	Analog
2	anaplastic astrocytoma	Anaplastic Astrocytoma
326	angiogenesis	Angiogenesis
1	angiogenic activity	Angiogenesis
2	angiopoietins	Angiopoietins
2	anti-angiogenic therapy	Antiangiogenic Therapy
19	antiangiogenic therapy	Antiangiogenic Therapy
3	antitumor-activity	Antitumor Activity
95	apoptosis	Apoptosis
2	arachidonic acid	Arachidonic Acid
7	astrocytes	Astrocytes
2	astrocytes induce	Astrocytes
2	astrocytoma	Astrocytoma
2	astrocytomas	Astrocytoma
71	barrier	Blood-Brain Barrier
13	barrier disruption	Blood-Brain Barrier Disruption
23	barrier permeability	Blood-Brain Barrier Permeability
4	basement-membrane	Basement-Membrane
3	bcnu	Carmustine
53	bevacizumab	Bevacizumab
3	bevacizumab plus irinotecan	Bevacizumab
16	binding	Binding
8	blood	Blood
5	blood brain	Blood-Brain Barrier
291	blood brain barrier	Blood-Brain Barrier
375	blood-brain barrier	Blood-Brain Barrier
9	blood-brain barrier disruption	Blood-Brain Barrier Disruption
2	blood-flow	Cerebral Blood Flow
20	blood-tumor barrier	Blood-Tumor Barrier
2	blood-vessels	Cerebral Blood Vessels
2	blood-volume	Cerebral Blood Volume
2	boron neutron capture therapy	Boron Neutron Capture Therapy
2	boronophenylalanine	Boronophenylalanine
4	bradykinin analog	Bradykinin Analog
4	bradykinin infusion	Bradykinin Infusion
106	brain	Brain

20	brain barrier	Blood-Brain Barrier
2	brain cyst	Brain Cyst
5	brain edema	Brain Edema
4	brain endothelial cells	Endothelial Cells
2	brain microvascular endothelium	Brain Microvascular Endothelium
3	brain neoplasms	Glioblastoma
106	brain tumor	Glioblastoma
96	brain tumors	Glioblastoma
6	brain tumour	Glioblastoma
43	brain-tumors	Glioblastoma
3	breast cancer	Glioblastoma
26	breast-cancer	Glioblastoma
3	c6 glioma	C6 Glioma
7	c6 glioma cells	C6 Glioma Cells
4	c6 glioma-cells	C6 Glioma Cells
298	cancer	Glioblastoma
19	cancer cells	Glioblastoma Cells
3	cancer resistance protein	Cancer Resistance Protein
3	cancer stem cells	Cancer Stem Cells
5	cancer-cells	Glioblastoma Cells
2	capillary	Capillary
2	capillary ultrastructure	Capillary Ultrastructure
5	carboplatin	Carboplatin
10	carcinoma	Glioblastoma
1	cat retina	Blood-Brain Barrier
3	cell	Cells
3	cell lung cancer	Cell Lung Cancer
4	cell-migration	Cell Migration
200	cells	Cells
87	central nervous system	Central Nervous System
2	central-nervous-system	Central Nervous System
2	cerebral blood flow	Cerebral Blood Flow
49	cerebral blood volume	Cerebral Blood Volume
2	cerebral gliomas	Glioblastoma
4	cerebrospinal fluid	Cerebrospinal Fluid
2	cereport rmp-7	Cereport RMP-7
125	chemotherapy	Chemotherapy
2	children	Children's Cancer Group
2	childrens cancer group	Children's Cancer Group
3	cisplatin	Cisplatin
15	classification	Classification
2	clinical implications	Clinical Implications
3	cns	Central Nervous System

2	coculture model	Coculture Model
18	combination	Combination
2	combination chemotherapy	Combination Chemotherapy
2	complex	Complex
3	computed tomography	Computed Tomography
2	computed-tomography	Computed Tomography
3	contrast	Contrast Agents
11	contrast agent	Contrast Agents
2	contrast agents	Contrast Agents
2	contrast enhanced mr	Contrast-Enhanced MRI
3	contrast enhancement	Contrast-Enhanced MRI
7	convection enhanced delivery	Convection-Enhanced Delivery
3	convection-enhanced delivery	Convection-Enhanced Delivery
2	ct	Computed Tomography
2	cyclophosphamide	Cyclophosphamide
2	cyclosporine a	Cyclosporine A
4	damage	Damage
4	death	Death
167	delivery	Delivery
4	density	Density
11	design	Design
4	dexamethasone	Dexamethasone
2	diagnosis	Diagnosis
47	differentiation	Differentiation
11	disease	Glioblastoma
107	disruption	Blood-Brain Barrier Disruption
3	down-regulation	down-regulation
47	doxorubicin	doxorubicin
10	drug	Drug
195	drug delivery	Drug Delivery
4	drug discovery	Drug Discovery
51	drug-delivery	Drug Delivery
2	drug-resistance	Drug Resistance
4	drugs	Drug
12	edema	Brain Edema
45	efficacy	Efficacy
2	endocannabinoid system	Endocannabinoid System
2	endothelial cell	Endothelial Cells
2	endothelial cell growth	Endothelial Growth Factor
141	endothelial cells	Endothelial Cells
210	endothelial growth factor	Endothelial Growth Factor
106	endothelial-cells	Endothelial Cells
2	enzymatic-activity	Enzymatic Activity

2	epidermal growth factor	Epidermal Growth Factor
2	epithelial-cells	Epithelial Cells
422	expression	Gene Expression
5	extracellular matrix	Extracellular Matrix
3	extracellular vesicles	Extracellular Vesicles
4	extracellular-matrix	Extracellular Matrix
2	f-18 fluorodeoxyglucose	F-18 Fluorodeoxyglucose
33	factor expression	Factor Expression
6	factor family	Factor Family
20	factor gene	Factor Gene
2	factor hgf	Hepatocyte Growth Factor
16	factor messenger rna	Messenger RNA
5	factor receptor	Factor Receptor
17	factor vegf	Vascular Endothelial Growth Factor
2	factor-alpha	Factor-alpha
3	factor-i	Factor-i
4	family	Factor Family
2	fdg-pet	Fdg-pet
3	features	Features
7	fibroblast growth factor	Fibroblast Growth Factor
9	flow	Cerebral Blood Flow
2	flt-1	Flt-1
2	focal adhesion kinase	Focal Adhesion Kinase
104	focused ultrasound	Focused Ultrasound
2	formyl peptide receptors	Formyl Peptide Receptors
2	formylpeptide receptor	Formyl Peptide Receptors
2	gelatinase-a	Gelatinase-A
26	gene	Gene
12	gene delivery	Gene Delivery
9	gene expression	Gene Expression
22	gene-expression	Gene Expression
4	gene-therapy	Gene Therapy
2	glial cells	Glial Cells
252	glioblastoma	Glioblastoma
8	glioblastoma (gbm)	Glioblastoma
108	glioblastoma multiforme	Glioblastoma
57	glioblastoma-multiforme	Glioblastoma
152	glioma	Glioblastoma
6	glioma cells	Glioblastoma Cells
48	glioma-cells	Glioblastoma Cells
80	gliomas	Glioblastoma
8	gold nanoparticles	Gold Nanoparticles
23	grade	Tumor Grade
165	growth	Tumor Growth

15	growth factor	Growth Factor
2	growth factors	Growth Factor
8	growth-factor	Growth Factor
2	guanylate cyclase	Guanylate Cyclase
5	health	Health
2	hemangioblastomas	Hemangioblastomas
9	high grade gliomas	Glioblastoma
3	high-grade glioma	Glioblastoma
2	high-grade gliomas	Glioblastoma
21	human brain tumors	Glioblastoma
3	human endothelial cells	Human Endothelial Cells
6	human glioblastoma cells	Human Glioblastoma Cells
6	human glioblastoma multiforme	Glioblastoma
4	human glioma	Human Glioblastoma
6	human glioma cells	Human Glioblastoma Cells
22	human gliomas	Human Glioblastoma
2	human keratinocytes	Human Keratinocytes
27	hypoxia	Hypoxia
26	identification	Identification
2	imaging proliferation	Imaging Proliferation
8	immunotherapy	Immunotherapy
2	in situ hybridization	In Situ Hybridization
114	in vitro	In Vitro
82	in vivo	In Vitro
99	in-vitro	In Vitro
118	in-vivo	In Vitro
2	increases	Increases
3	induced apoptosis	Apoptosis
3	induced transcriptional activation	Induced Transcriptional Activation
33	induction	Activation
14	inflammation	Inflammation
115	inhibition	Inhibition
7	inhibitor	Inhibitors
3	inhibitors	Inhibitors
7	injury	Injury
2	intercellular communication	Intercellular Communication
2	interstitial fluid pressure	Interstitial Fluid Pressure
3	intra-arterial chemotherapy	Intra-Arterial Chemotherapy
5	intraarterial chemotherapy	Intra-Arterial Chemotherapy
27	intracarotid infusion	Intracarotid Infusion
43	invasion	Invasion

6	invitro	In Vitro
11	invivo	In Vitro
5	involvement	Involvement
3	ion channels	Ion Channels
3	ionizing radiation	Ionizing Radiation
8	iron oxide nanoparticles	Iron Oxide Nanoparticles
2	irradiation	Irradiation
2	ischemic stroke	Ischemic Stroke
4	iv collagenase	IV Collagenase
6	kinase	Kinase
2	kinetics	Kinetics
2	leakage space	Leakage Space
2	line	Line
10	liposm	Liposome
14	liposomal doxorubicin	Liposomal Doxorubicin
10	localization	Localization
9	macrophages	Macrophages
15	magnetic resonance imaging	Magnetic Resonance Imaging
120	malignant glioma	Glioblastoma
77	malignant gliomas	Glioblastoma
2	mammalian heparanase	Mammalian Heparanase
5	matrix metalloproteinases	Matrix Metalloproteinases
2	mechanism	Mechanisms
70	mechanisms	Mechanisms
2	meningiomas	Glioblastoma
4	mesoporous silica nanoparticles	Mesoporous Silica Nanoparticles
2	messenger rna expression	Messenger RNA Expression
25	messenger-rna	Messenger RNA
4	metabolism	Metabolism
3	metastases	Metastases
15	metastasis	Metastases
2	methionine uptake	Methionine Uptake
2	methotrexate	Methotrexate
4	methotrexate delivery	Methotrexate Delivery
4	mice	Mice
7	microbubbles	Microbubbles
4	microenvironment	Tumor Microenvironment
4	microvascular endothelial cells	Microvascular Endothelial Cells
24	microvascular permeability	Microvascular Permeability
2	microvessel density	Microvessel Density
19	migration	Cell Migration
9	mitogen	Mitogen

88	model	Model
28	modulation	Modulation
2	monoclonal antibodies	Monoclonal Antibodies
10	monoclonal antibody	Monoclonal Antibodies
9	monoclonal-antibody	Monoclonal Antibodies
2	monolayers	Monolayers
2	mouse	Mice
3	mouse model	Mouse Model
2	mr	MR Imaging
2	mr imaging	MR Imaging
32	mri	MR Imaging
1	muller cells	Muller Cells
7	multidrug resistance	Multidrug Resistance
3	multidrug-resistance	Multidrug Resistance
2	multiple sclerosis	Multiple Sclerosis
2	mutant	Mutations
4	mutations	Mutations
116	nanoparticles	Nanoparticles
5	necrosis factor alpha	Necrosis Factor Alpha
4	nervous-system	Nervous System
2	neurons	Neurons
6	neutron capture therapy	Neutron Capture Therapy
9	newly diagnosed glioblastoma	Newly Diagnosed Glioblastoma
7	nf kappa b	NF-κB
2	nf-kappa-b	NF-κB
15	nitric oxide	Nitric Oxide
16	nitric oxide synthase	Nitric Oxide Synthase
2	nitric oxide synthases	Nitric Oxide Synthase
7	nitric-oxide	Nitric Oxide
2	normal rat brain	Normal Rat Brain
5	occludin	Occludin
1	optic-nerve	Optic Nerve
12	oxidative stress	Oxidative Stress
1	oxygen	Oxygen
15	p glycoprotein	P-Glycoprotein
7	p-glycoprotein	P-Glycoprotein
11	paclitaxel	Paclitaxel
4	parameters	Parameters
20	pathway	Pathway
2	pdgf-b	PDGF-B
4	pegylated liposomal doxorubicin	Pegylated Liposomal Doxorubicin
15	penetration	Penetration

5	peptide	Peptides
3	peptides	Peptides
18	perfusion	Perfusion
7	pericytes	Pericytes
240	permeability	Vascular Permeability
67	permeability factor	Vascular Permeability Factor
15	pharmacokinetics	Pharmacokinetics
4	phase ii trial	Phase II Trial
2	phase-i	Phase I Trial
12	phase-ii	Phase II Trial
2	phosphatase-activity	Phosphatase Activity
5	phosphorylation	Phosphorylation
5	photodynamic therapy	Photodynamic Therapy
4	photothermal therapy	Photothermal Therapy
2	plus irinotecan	Plus Irinotecan
2	polysorbate 80	Polysorbate 80
22	positron emission tomography	Positron Emission Tomography
2	potassium channels	Potassium Channels
25	progression	Tumor Progression
70	proliferation	Proliferation
3	promotes	Promotes
46	protein	Proteins
9	protein kinase c	Protein Kinase C
2	protein product	Protein Product
3	proteins	Proteins
2	purification	Purification
2	quantification	Quantification
11	radiation	Radiation
4	radiation therapy	Radiation Therapy
11	radiation-therapy	Radiation Therapy
77	radiotherapy	Radiation Therapy
15	rat	Albino Rat
3	rat brain	Rat Glioma
8	rat glioma	Rat Glioma
2	rat glioma model	Rat Glioma
17	rat-brain	Rat Glioma
2	rb-82	Rb-82
57	receptor	Receptor
4	receptor tyrosine kinase	Receptor Tyrosine Kinase
2	receptor tyrosine kinases	Receptor Tyrosine Kinase
13	receptors	Receptor
6	recurrent glioblastoma	Recurrent Glioblastoma
2	recurrent glioma	Recurrent Glioblastoma

2	recurrent malignant glioma	Recurrent Glioblastoma
3	regression	Regression
4	release	Release
4	resection	Resection
53	resistance	Drug Resistance
2	rmp-7	RMP-7
2	sequence	Sequence
3	signal transduction	Signal Transduction
2	signal-transduction	Signal Transduction
9	size	Size
7	smooth muscle cells	Smooth Muscle Cells
3	sodium borocaptate	Sodium Borocaptate
4	solid lipid nanoparticles	Solid Lipid Nanoparticles
7	solid tumors	Glioblastoma
2	solitary metastases	Solitary Metastases
4	sonodynamic therapy	Sonodynamic Therapy
2	spinal cord	Spinal Cord
14	stem cells	Stem Cells
7	stem-cells	Stem Cells
16	strategy	Strategy
2	sulfate proteoglycans	Sulfate Proteoglycans
2	suppressor gene	Tumor Suppressor Gene
110	survival	Survival
2	synchrotron radiation	Synchrotron Radiation
2	synthase	Synthase
40	system	System
4	target	Target
22	targeted delivery	Targeted Delivery
142	temozolomide	Adjuvant Temozolomide
2	therapeutic implications	Therapeutic Implications
3	therapeutic target	Therapeutic Target
164	therapy	Therapy
2	thymidine kinase 1	Thymidine Kinase 1
11	tight junction	Tight Junctions
29	tight junctions	Tight Junctions
8	tissue	Tissue
2	tl 201 spect	Tl-201 SPECT
2	tnf-alpha	TNF-Alpha
2	to tumor transport	Tumor-Specific Delivery
2	tomography	Tomography
2	toxicity	Toxicity
2	tracer	Tracer
3	transcription factor	Transcription Factor
2	transduction	Transduction

2	transfer constants	Transfer Constants
3	transferrin	Transferrin
2	transferrin receptor	Transferrin Receptor
67	transport	Transport
4	transporters	Transporters
3	trial	Trial
73	tumor	Glioblastoma
74	tumor angiogenesis	Angiogenesis
3	tumor grade	Tumor Grade
10	tumor growth	Tumor Growth
13	tumor microenvironment	Tumor Microenvironment
3	tumor necrosis factor	Tumor Necrosis Factor
2	tumor progression	Tumor Progression
2	tumor suppressor gene	Tumor Suppressor Gene
4	tumor-cells	Glioblastoma Cells
31	tumor-growth	Tumor Growth
2	tumorigenicity	Tumorigenicity
145	tumors	Glioblastoma
20	tyrosine kinase	Tyrosine Kinase
3	tyrosine phosphorylation	Tyrosine Phosphorylation
2	up regulation	Up-Regulation
31	up-regulation	Up-Regulation
2	vanilloid receptors	Vanilloid Receptors
61	vascular endothelial growth factor	Vascular Endothelial Growth Factor
59	vascular permeability	Vascular Permeability
39	vascular permeability factor	Vascular Permeability Factor
49	vascular-permeability	Vascular Permeability
2	vascular-permeability factor	Vascular Permeability Factor
5	vasculature	Vasculature
36	vegf	Vascular Endothelial Growth Factor
2	vegf receptor	VEGF Receptors
2	vegf receptors	VEGF Receptors
2	verapamil	Verapamil
2	vessels	Vessels
4	vivo	In Vitro
3	volume	Cerebral Blood Volume
2	water channel	Water Channel
2	xenografts	Xenografts

Supplementary Table S2. Examples of merging synonymous terms and the reference standards used for normalization
