# Supplementary material for: Scientometric analysis of glioblastoma and blood-brain barrier research (1995−2024): evolving trends and therapeutic challenges
Source: Front Oncol. 2025 Sep 25;15:1649414. doi: 10.3389/fonc.2025.1649414 (PMC12507556; doi:10.3389/fonc.2025.1649414)
Supplement: Supplementary file 5 [file DataSheet5.pdf]

| Organization | Documents | Citations | Total link strength |
| --- | --- | --- | --- |
| Harvard University | 71 | 13564 | 80 |
| Massachusetts General Hospital | 44 | 10728 | 80 |
| Mayo Clinic | 36 | 8479 | 50 |
| University of Texas MD Anderson Cancer Center | 46 | 7798 | 65 |
| Cleveland Clinic | 20 | 6641 | 30 |
| University of California, San Francisco | 50 | 6457 | 52 |
| Duke University | 38 | 6403 | 34 |
| University of California, Los Angeles | 35 | 5324 | 39 |
| Emory University | 39 | 5248 | 32 |
| King's College London | 15 | 5205 | 3 |
| University of Michigan | 25 | 5012 | 21 |
| Memorial Sloan Kettering Cancer Center | 30 | 4839 | 15 |
| Brigham and Women’s Hospital | 21 | 4348 | 40 |
| National Cancer Institute (NCI) | 47 | 4161 | 65 |
| Fudan University | 16 | 4087 | 28 |
| University of Toronto | 39 | 3965 | 35 |
| Ohio State University | 29 | 3488 | 35 |
| Henry Ford Hospital | 16 | 3446 | 18 |
| New York University (NYU) | 25 | 3366 | 13 |
| Heidelberg University | 16 | 3348 | 26 |
| Harvard Medical School | 49 | 3196 | 65 |
| Massachusetts Institute of Technology (MIT) | 16 | 3186 | 25 |
| Johns Hopkins University | 49 | 3133 | 17 |
| Goethe University Frankfurt | 16 | 3117 | 13 |
| Chinese Academy of Sciences | 52 | 2934 | 34 |
| University of Virginia | 23 | 2896 | 28 |
| University of Maryland | 21 | 2526 | 16 |
| University of Tübingen | 18 | 2476 | 4 |
| University of California, San Diego | 27 | 2461 | 20 |
| China Medical University | 79 | 2460 | 28 |
| Chang Gung University | 26 | 2423 | 8 |
| University of Minnesota | 24 | 2121 | 18 |
| University of Freiburg | 21 | 2017 | 4 |
| University of Alabama at Birmingham | 16 | 1715 | 12 |
| [illegible]M (French National Institute of Health and Medical Re[illegible] | 27 | 1710 | 15 |
| University of Pennsylvania (Penn) | 28 | 1703 | 21 |
| Dana-Farber Cancer Institute | 50 | 1698 | 60 |
| Northwestern University | 24 | 1578 | 25 |
| Yale University | 16 | 1541 | 4 |
| University College London (UCL) | 19 | 1497 | 11 |
| University of Paris 05 | 18 | 1484 | 11 |
| Hospital for Sick Children | 18 | 1470 | 22 |
| Sichuan University | 30 | 1416 | 25 |
| [illegible]NRS (French National Centre for Scientific Research | 16 | 1392 | 9 |
| Third Military Medical University | 19 | 1375 | 13 |
| University of Illinois | 29 | 1323 | 7 |
| German Cancer Research Center (DKFZ) | 29 | 1303 | 24 |
| University of Milan | 18 | 1223 | 9 |
| Tufts University | 19 | 1188 | 18 |

| | | | |
|---|---|---|---|
| Seoul National University | 18 | 1109 | 2 |
| Stanford University | 27 | 1085 | 26 |
| National Taiwan University | 20 | 1079 | 15 |
| Washington University | 27 | 1046 | 12 |
| Alkermes Inc. | 16 | 993 | 19 |
| Wayne State University | 17 | 987 | 12 |
| Shanghai Jiao Tong University | 15 | 981 | 17 |
| Columbia University | 23 | 957 | 10 |
| Capital Medical University | 24 | 948 | 11 |
| University of Pittsburgh | 21 | 936 | 25 |
| Oregon Health and Science University | 22 | 903 | 18 |
| Nanjing Medical University | 19 | 845 | 23 |
| Cedars-Sinai Medical Center | 23 | 766 | 6 |
| National Yang Ming University | 20 | 765 | 4 |
| Sun Yat-sen University | 27 | 729 | 16 |
| University of Zurich | 16 | 718 | 14 |
| University of Washington | 15 | 703 | 17 |
| Peking University | 15 | 698 | 8 |
| Soochow University | 21 | 680 | 17 |
| University of California, Irvine | 17 | 678 | 11 |
| Henan University | 15 | 667 | 7 |
| National Institutes of Health (NIH) | 15 | 628 | 21 |
| Uppsala University | 19 | 589 | 13 |
| Baylor College of Medicine | 16 | 561 | 11 |
| nal Institute of Neurological Disorders and Stroke (NI | 15 | 554 | 20 |
| Tsinghua University | 20 | 554 | 8 |
| Fourth Military Medical University | 15 | 548 | 4 |
| Southern Medical University | 22 | 508 | 10 |
| China Pharmaceutical University | 19 | 474 | 5 |
| University of Florida | 19 | 464 | 5 |
| Huazhong University of Science and Technology | 22 | 451 | 14 |
| Russian Academy of Sciences | 19 | 450 | 5 |
| Shandong University | 22 | 439 | 7 |
| Taipei Medical University | 16 | 428 | 11 |
| ꞆNRS (French National Centre for Scientific Research | 15 | 422 | 2 |
| Zhejiang University | 27 | 414 | 14 |
| Tianjin Medical University | 15 | 411 | 7 |
| Key Laboratory of Neurooncology, Liaoning Province | 15 | 359 | 15 |
| Harbin Medical University | 15 | 325 | 5 |
| Aix-Marseille University | 19 | 281 | 6 |
| Zhengzhou University | 15 | 220 | 7 |

**Supplementary Table S3.** Institutional Co-authorship Network Data
